# National prevalence of atopic dermatitis in Korean adolescents from 2009 to 2022

**DOI:** 10.1038/s41598-024-62475-4

**Published:** 2024-05-29

**Authors:** Mafaz Kattih, Hojae Lee, Hyesu Jo, Jinyoung Jeong, Hyejun Kim, Jaeyu Park, Hwi Yang, Ann Nguyen, Hyeon Jin Kim, Hyeri Lee, Minji Kim, Myeongcheol Lee, Rosie Kwon, Sunyoung Kim, Ai Koyanagi, Min Seo Kim, Masoud Rahmati, Guillermo F. López Sánchez, Elena Dragioti, Ju Hee Kim, Selin Woo, Seong H. Cho, Lee Smith, Dong Keon Yon

**Affiliations:** 1 Department of Medicine, University of South Florida Morsani College of Medicine, Tampa, FL USA; 2 Center for Digital Health, Medical Science Research Institute, Kyung Hee University College of Medicine, Seoul, South Korea; 3 Department of Regulatory Science, Kyung Hee University, Seoul, South Korea; 4 Department of Medicine, Kyung Hee University College of Medicine, Seoul, South Korea; 5 Department of Applied Information Engineering, Yonsei University, Seoul, South Korea; 6 Department of Family Medicine, Kyung Hee University Medical Center, Kyung Hee University College of Medicine, Seoul, South Korea; 7 Research and Development Unit, Parc Sanitari Sant Joan de Deu, Barcelona, Spain; 8 Cardiovascular Disease Initiative, Broad Institute of MIT and Harvard, Cambridge, MA USA; 9 Health Service Research and Quality of Life Center (CEReSS), Assistance Publique-Hôpitaux de Marseille, Aix-Marseille Université, Marseille, France; 10 Department of Physical Education and Sport Sciences, Faculty of Literature and Human Sciences, Lorestan University, Khoramabad, Iran; 11 Department of Physical Education and Sport Sciences, Faculty of Literature and Humanities, Vali-E-Asr University of Rafsanjan, Rafsanjan, Iran; 12 Division of Preventive Medicine and Public Health, Department of Public Health Sciences, School of Medicine, University of Murcia, Murcia, Spain; 13 Department of Medical and Health Sciences, Pain and Rehabilitation Centre, Linköping University, Linköping, Sweden; 14 Research Laboratory Psychology of Patients, Families, and Health Professionals, Department of Nursing, School of Health Sciences, University of Ioannina, Ioannina, Greece; 15 Department of Pediatrics, Kyung Hee University Medical Center, Kyung Hee University College of Medicine, 23 Kyungheedae-ro, Dongdaemun-gu, Seoul, 02447 South Korea; 16 Division of Allergy and Immunology, Department of Internal Medicine, USF Morsani College of Medicine, Tampa, FL USA; 17 Centre for Health, Performance and Wellbeing, Anglia Ruskin University, Cambridge, CB1 1PT UK

**Keywords:** Atopic dermatitis, Prevalence, Adolescents, South Korea, Health care, Medical research, Risk factors

## Abstract

Previous studies have examined the prevalence of allergic diseases in adolescents 1–2 years after the emergence of the COVID-19 pandemic. However, more data is needed to understand the long-term impact of COVID-19 on allergic diseases. Thus, we aimed to examine the trend of the atopic dermatitis prevalence in Korean adolescents before and during the COVID-19 pandemic across 14 years. Additionally, we analyze the risk factors of atopic dermatitis (AD) based on the results. The Korean Disease Control and Prevention Agency conducted the Korea Youth Risk Behavior Web-based Survey from 2009 to 2022, from which the data for this study were obtained. Prevalence trends were compared across subgroups, and the β difference (β_diff_) was calculated. We computed odds ratios to examine changes in the disease prevalence before and during the pandemic. This study included a total of 917,461 participants from 2009 to 2022. The prevalence of atopic dermatitis increased from 6.79% (95% CI 6.66–6.91) in 2009–2011 to 6.89% (95% CI 6.72–7.05) in 2018–2019, then decreased slightly to 5.82% (95% CI 5.60–6.04) in 2022. Across the 14 years, middle school student status, low parent’s highest education level, low household income, non-alcohol consumption, non-smoker smoking status, no suicidal thoughts, and no suicide attempts were associated with increased risk of atopic dermatitis, while female sex, rural residence, high BMI, low school performance, low household income, and no feelings of sadness and despair was associated with a small increase. This study examined the prevalence of atopic dermatitis across an 18-year, and found that the prevalence increased in the pre-pandemic then decreased during the start of the pandemic and remained constant throughout the pandemic. This trend could be explained mainly by the large scale social and political changes that occurred during the COVID-19 pandemic.

## Introduction

The COVID-19 pandemic has brought about changes in various aspects, including daily life and hospital treatment. Furthermore, the South Korean government recommended the use of masks not only for individuals exhibiting respiratory symptoms but also for healthy members of the general public as part of a nationwide effort to prevent and control the spread of infection. Such changes brought about by COVID-19 may have also impacted the prevalence and condition of skin-related diseases^1^. Therefore, it is necessary to compare the prevalence of skin-related ailments before and after the COVID-19 pandemic, as well as to analyze prevalence rates during the period of the pandemic^2,3^. For this reason, it is imperative to focus special attention on the changes in specific diseases, particularly atopic dermatitis (AD), following COVID-19. The prolonged exposure of the skin to warm and humid conditions, as experienced with mask wearing, can exacerbate AD. Among the diseases that have undergone changes after COVID-19, we are particularly interested in AD.

AD is a widely encountered condition, affecting 15–20% of adolescents^1,4–6^. Additionally, although AD is not a fatal disease with a low mortality rate, it can recur and has a negative impact on people’s health and quality of life^7,8^. Therefore, there is a need for further research about AD including COVID-19 pandemic. However, there has been a lack of studies analyzing and interpreting the prevalence of AD up to 2022. Since the COVID-19 pandemic occurred over a short period starting from 2020, it is essential to conduct detailed analysis including the most recent data^9–13^. Additionally, whereas previous studies lacked investigation into risk factors or their utilization of invalidated definitions of AD such as claim-based codes^11^, we identify risk factors influencing AD by analyzing the data including information up to 2022.

Therefore, our study aims to analyze the changing prevalence of AD before and after pandemic and to conduct an analysis considering the changed situation in 2022, including the risk factors. Based on this analysis, we aim to identify risk factors that influence AD and present a new perspective on AD after 2022, including aspects that have not been considered.

## Methods

### Patient selection and data collection

In this study, we used data from the Korea Youth Risk Behavior Web-based Survey (KYRBS) from 2009 to 2022^14,15^. The KYRBS is an online survey administered to Korean adolescents in computer laboratories at their schools, and it utilizes a self-reporting method that monitors the health and behavior of Korean youths while being supervised by the Korean Disease Control and Prevention Agency (KDCA)^9,16^. This data includes information about behaviors and health such as smoking, drinking, physical activity, and allergic diseases. To ensure that the survey data reflects the entire Korean population, it employs sample selection methods such as stratified cluster sampling and two-step stratification^16,17^. And the weighting is calculated by multiplying the inverse of the sampling rate by the inverse of the response rate, and then multiplying this by the post-adjustment weighting rate^16^. The data collected included all adolescents aged 13–18 years with a response rate over 95% while participants with missing data were excluded. The research protocol received approval from both the Institutional Review Board of Kyung Hee University (KHUH 2022-06-042) and the KDCA. Written informed consent was obtained from all participants prior to their involvement in the study. Additionally, the KYRBS provides public access to its data, making it a valuable resource for conducting various epidemiological investigations. This research adhered to the ethical guidelines established by relevant national, and institutional review boards for human research and followed the 1975 Helsinki Declaration, as amended in 2008.

### Ascertainment of AD

Our research aimed to confirm the trend in the prevalence of AD among adolescents from 2009 to 2022^16,18^. Additionally, the objective of our study was to investigate the risk factors associated with atopic dermatitis. Patients with AD were defined by positive responses to two questions: “Have you ever been diagnosed AD by a doctor within the past 12 months?” and “Have you ever been diagnosed AD by a doctor throughout life?” In addition, the questions were used the same during the study^11,19,20^.

### Covariates

The data was divided into time periods before and during the COVID-19 pandemic. Since the first case of COVID-19 in South Korea was reported in January 2020, the year 2020 was defined as the first year of the pandemic^21,22^. Before COVID-19, 2009–2019, was subdivided into intervals of 2 to 3 years to obtain a stable estimate of the prevalence of allergic diseases (2009 to 2011, 2012–2013, 2014–2015, 2016–2017, and 2018–2019). Prevalence of AD was further by stratified variables including grade, sex, region of residence (rural and urban)^23–28^, body mass index (BMI; underweight, normal, overweight, obese, and unknown), school performance (low, lower-middle, middle, upper-middle, and high), parent’s highest educational level (middle school graduated or under, high school graduated, university graduated or higher, and unknown), household income (lower or middle lower, middle, and middle higher or higher), alcohol consumption (non-drinker, 1–2, 3–5, 6–9, and ≥ 10 day/month), smoking status (non-smoker and smoker), stress status (none, mild, moderate, high, and severe), sadness and despair, suicidal thoughts, and suicide attempts. BMI was calculated with body height, weight, sex, and age with reference to the 2017 Korean National Growth Charts for children and adolescents^29^.

### Statistical analysis

We included demographic characteristics of the study participants as covariates^30^. We analyzed secondary data from KYRBS spanning a total of 14 years, from 2009 to 2022. Only records with complete data were included in the study. Analysis was conducted using composite sampling linear and logistic regression models. The term “composite sampling linear regression” refers to a method of conducting linear regression analysis by amalgamating data obtained from multiple sampling sources^31^. To investigate the trends in prevalence before and during the COVID-19 pandemic, we utilized a weighted linear regression model, deriving a β coefficient along with a 95% confidence interval (CI)^32^. The β_diff_ was used to emphasize the difference between the pre-pandemic (2009–2020) and the pandemic (2020 and 2022). This method was chosen to examine changes over time and to contrast the effects before and during the pandemic. We used a binary logistic regression model to calculate the odds ratio for the 2009–2019 and 2020–2022. We also employed this method to identify risk factors of AD and calculate the ratio of odds ratio (OR) by using odds ratios from before and during the pandemic. We further determined risk factors for the years 2020, 2021 and 2022, to deepen our understanding of the pandemic’s impact^33–35^. Categorical data were presented as frequencies and percentages, whereas numerical data were presented as means along with their corresponding 95% CIs. All statistical analyses were performed using SAS version 9.4 software (SAS Institute, Cary, NC, USA) and GraphPad Prism version 9.5.0 (GraphPad, San Diego, CA, USA)^35–37^.

### Ethics approval

The study protocol was approved by the Institutional Review Board of KDCA (2014-06EXP-02-P-A).

### Informed consent

All participants provided written informed consent.

## Results

**Figure 1:**
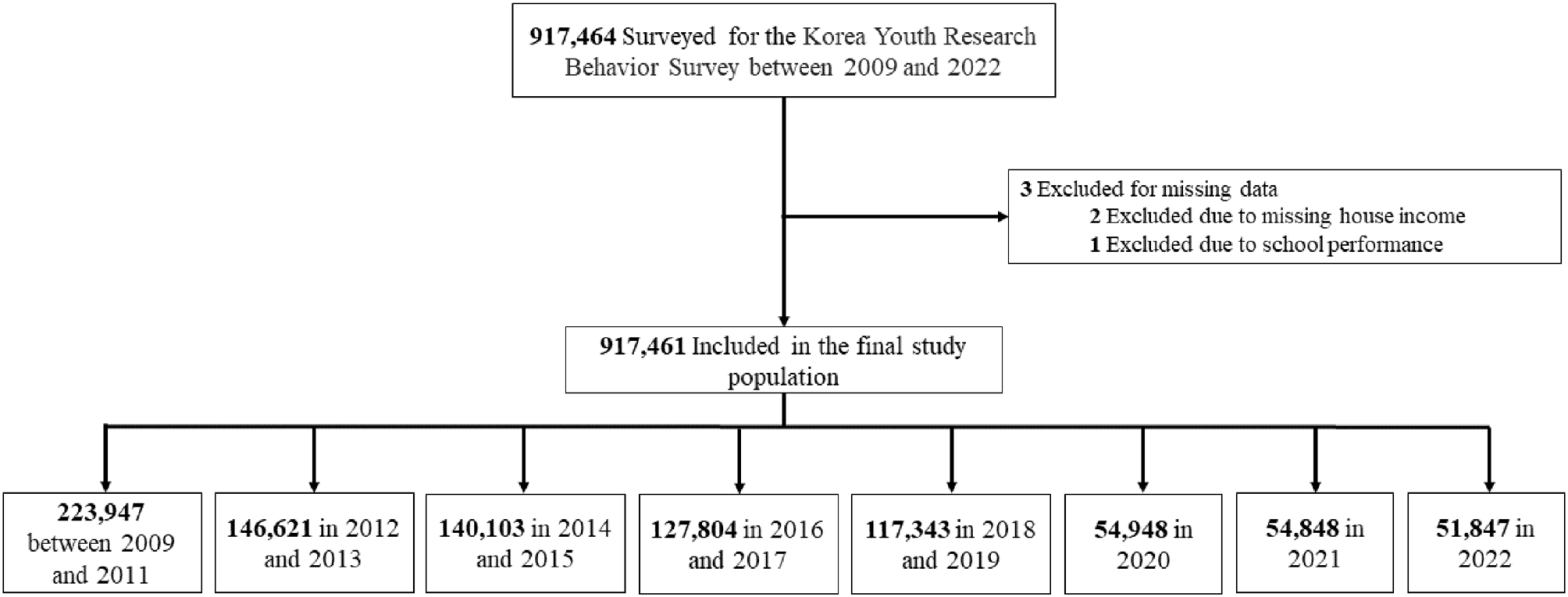
Study population in KYRBS, 2009–2022. *KYRBS* Korea Youth Risk Behavior Web-based Survey.

Table 1, Tables S1 and S2 show the data collected from KYRBS. The survey included a total of 917,461 participants aged 13–18 years from 2009 to 2022. The number of participants in each period was as follows: 223,947 from 2009 to 2011, 146,621 from 2012 to 2013, 140,103 from 2014 to 2015, 127,804 from 2016 to 2017, and 117,343 from 2018 to 2019 (Fig. 1). During the pandemic, there were 54,948 participants in 2020, 54,848 in 2021, and 51,847 in 2022. In terms of participant demographics, 471,306 (51.37%) of the participants were male, 446,155 (48.63%) female, and 502,419 (54.76%) lived in rural areas. 106,900 (11.65%) were people with obese, 765,939 (83.48%) were non-drinkers and 749,053 (81.64%) non-smokers. 30,765 (3.35%) of participants reported suicide attempts while 137,537 (14.99%) reported suicidal thoughts.

**Table 1:** Baseline characteristics of participating adolescents in KYRBS, 2009–2022 (n = 917,461).

Characteristics	Total	Pre-pandemic (2009–2019)					Pandemic (2020–2022)		
Year	2009–2022	2009–2011	2012–2013	2014–2015	2016–2017	2018–2019	2020	2021	2022
Overall, n	917,461	223,947	146,621	140,103	127,804	117,343	54,948	54,848	51,847
Grade, n (%)									
7th–9th grade (middle school)	468,443 (51.06)	114,453 (51.11)	73,827 (50.35)	70,455 (50.29)	63,104 (49.38)	59,613 (50.80)	28,961 (52.71)	30,015 (54.72)	28,015 (54.03)
10th–12th grade (high school)	449,018 (48.94)	109,494 (48.89)	72,794 (49.65)	69,648 (49.71)	64,700 (50.62)	57,730 (49.20)	25,987 (47.29)	24,833 (45.28)	23,832 (45.97)
Sex, n (%)									
Male	471,306 (51.37)	115,876 (51.74)	74,876 (51.07)	71,674 (51.16)	65,427 (51.19)	60,304 (51.39)	28,353 (51.60)	28,401 (51.78)	26,395 (50.91)
Female	446,155 (48.63)	108,071 (48.26)	71,745 (48.93)	68,429 (48.84)	62,377 (48.81)	57,039 (48.61)	26,595 (48.40)	26,447 (48.22)	25,452 (49.09)
Region of residence, n (%)									
Rural	502,419 (54.76)	115,403 (51.53)	80,513 (54.91)	78,072 (55.72)	71,129 (55.65)	65,354 (55.69)	31,327 (57.01)	30,986 (56.49)	29,635 (57.16)
Urban	415,042 (45.24)	108,544 (48.47)	66,108 (45.09)	62,031 (44.28)	56,675 (44.35)	51,989 (44.31)	23,621 (42.99)	23,862 (43.51)	22,212 (42.84)
BMI group, n (%)^a^									
Underweight	214,927 (23.43)	58,085 (25.94)	36,181 (24.68)	33,365 (23.81)	27,705 (21.68)	24,725 (21.07)	11,342 (20.64)	11,563 (21.08)	11,961 (23.07)
Normal	465,651 (50.75)	118,022 (52.70)	77,389 (52.78)	72,825 (51.98)	64,673 (50.60)	57,972 (49.40)	25,798 (46.95)	25,189 (45.93)	23,783 (45.87)
Overweight	103,463 (11.28)	22,332 (9.97)	15,544 (10.60)	15,698 (11.20)	15,232 (11.92)	14,211 (12.11)	7279 (13.25)	6926 (12.63)	6241 (12.04)
Obese	106,900 (11.65)	18,243 (8.15)	13,469 (9.19)	14,139 (10.09)	16,523 (12.93)	17,176 (14.64)	9115 (16.59)	9767 (17.81)	8468 (16.33)
Unknown	26,520 (2.89)	7265 (3.24)	4038 (2.75)	4076 (2.91)	3671 (2.87)	3259 (2.78)	1414 (2.57)	1403 (2.56)	1394 (2.69)
School performance, n (%)									
Low (0–19 percentile)	102,336 (11.15)	28,013 (12.51)	19,054 (13.00)	15,278 (10.90)	12,711 (9.95)	11,401 (9.72)	5533 (10.07)	5413 (9.87)	4933 (9.51)
Lower-middle (20–39 percentile)	218,304 (23.79)	57,177 (25.53)	37,048 (25.27)	33,298 (23.77)	28,894 (22.61)	25,819 (22.00)	12,684 (23.08)	12,004 (21.89)	11,380 (21.95)
Middle (40–59 percentile)	259,529 (28.29)	60,245 (26.90)	40,002 (27.28)	39,114 (27.92)	36,436 (28.51)	34,760 (29.62)	16,585 (30.18)	16,903 (30.82)	15,484 (29.86)
Upper-middle (60–79 percentile)	225,194 (24.55)	53,428 (23.86)	34,655 (23.64)	34,949 (24.95)	32,546 (25.47)	29,647 (25.27)	13,410 (24.40)	13,444 (24.51)	13,115 (25.30)
High (80–100 percentile)	112,098 (12.22)	25,084 (11.20)	15,862 (10.82)	17,464 (12.47)	17,217 (13.47)	15,716 (13.39)	6736 (12.26)	7084 (12.92)	6935 (13.38)
Parent’s highest educational level, n (%)									
Middle school graduated or under	173,256 (18.88)	26,039 (11.63)	18,643 (12.72)	20,398 (14.56)	19,169 (15.00)	34,497 (29.40)	18,784 (34.19)	19,347 (35.27)	16,379 (31.59)
High school graduated	17,879 (1.95)	8713 (3.89)	3557 (2.43)	2,201 (1.57)	1451 (1.14)	931 (0.79)	368 (0.67)	318 (0.58)	340 (0.66)
University graduated or higher	253,762 (27.66)	85,045 (37.98)	50,637 (34.54)	40,313 (28.77)	32,677 (25.57)	21,345 (18.19)	8795 (16.01)	7853 (14.32)	7,097 (13.69)
Unknown	472,564 (51.51)	104,150 (46.51)	73,784 (50.32)	77,191 (55.10)	74,507 (58.30)	60,570 (51.62)	27,001 (49.14)	27,330 (49.83)	28,031 (54.06)
Household income, n (%)									
Middle lower or lower	165,287 (18.02)	54,730 (24.44)	32,214 (21.97)	24,626 (17.58)	19,120 (14.96)	15,366 (13.09)	7212 (13.13)	6203 (11.31)	5816 (11.22)
Middle	434,364 (47.34)	105,464 (47.09)	69,378 (47.32)	67,002 (47.82)	59,638 (46.66)	55,265 (47.10)	26,397 (48.04)	27,077 (49.37)	24,143 (46.57)
Middle higher or higher	317,810 (34.64)	63,753 (28.47)	45,029 (30.71)	48,475 (34.60)	49,046 (38.38)	46,712 (39.81)	21,339 (38.83)	21,568 (39.32)	21,888 (42.22)
Alcohol consumption, n (%)									
Non-drinker	765,939 (83.48)	176,814 (78.95)	120,332 (82.07)	117,470 (83.85)	108,778 (85.11)	99,276 (84.60)	49,056 (89.28)	49,045 (89.42)	45,168 (87.12)
1–2 day	87,842 (9.57)	26,530 (11.85)	15,117 (10.31)	13,220 (9.44)	11,395 (8.92)	10,387 (8.85)	3495 (6.36)	3532 (6.44)	4166 (8.04)
3–5 day	28,517 (3.11)	8461 (3.78)	5139 (3.51)	4410 (3.15)	3741 (2.93)	3571 (3.04)	1059 (1.93)	1010 (1.84)	1126 (2.17)
6–9 day	16,184 (1.76)	5427 (2.42)	2807 (1.91)	2332 (1.66)	1792 (1.40)	1914 (1.63)	637 (1.16)	589 (1.07)	686 (1.32)
≥ 10 day	18,979 (2.07)	6715 (3.00)	3226 (2.20)	2671 (1.91)	2098 (1.64)	2195 (1.87)	701 (1.28)	672 (1.23)	701 (1.35)
Smoking status, n (%)									
Non-smoker	749,053 (81.64)	163,808 (73.15)	112,728 (76.88)	114,508 (81.73)	110,143 (86.18)	101,727 (86.69)	49,318 (89.75)	49,519 (90.28)	47,302 (91.23)
Smoker	168,408 (18.36)	60,139 (26.85)	33,893 (23.12)	25,595 (18.27)	17,661 (13.82)	15,616 (13.31)	5630 (10.25)	5329 (9.72)	4545 (8.77)
Stress status, n (%)^b^									
None	28,795 (3.14)	5043 (2.25)	4129 (2.82)	5108 (3.65)	4906 (3.84)	4302 (3.67)	2018 (3.67)	1792 (3.27)	1497 (2.89)
Mild	139,224 (15.17)	30,329 (13.54)	20,691 (14.11)	23,503 (16.78)	21,001 (16.43)	17,910 (15.26)	9889 (18.00)	8585 (15.65)	7316 (14.11)
Moderate	384,391 (41.90)	91,742 (40.97)	60,243 (41.09)	60,830 (43.42)	54,292 (42.48)	48,041 (40.94)	24,379 (44.37)	23,226 (42.35)	21,638 (41.73)
High	262,510 (28.61)	68,284 (30.49)	44,238 (30.17)	37,647 (26.87)	34,584 (27.06)	33,391 (28.46)	14,059 (25.59)	15,254 (27.81)	15,053 (29.03)
Severe	102,541 (11.18)	28,549 (12.75)	17,320 (11.81)	13,015 (9.29)	13,021 (10.19)	13,699 (11.67)	4603 (8.38)	5991 (10.92)	6343 (12.23)
Sadness and despair, n (%)									
No	648,540 (70.69)	143,140 (63.92)	101,446 (69.19)	105,035 (74.97)	95,657 (74.85)	85,107 (72.53)	41,108 (74.81)	40,156 (73.21)	36,891 (71.15)
Yes	268,921 (29.31)	80,807 (36.08)	45,175 (30.81)	35,068 (25.03)	32,147 (25.15)	32,236 (27.47)	13,840 (25.19)	14,692 (26.79)	14,956 (28.85)
Suicidal thoughts, n (%)									
No	779,924 (85.01)	180,603 (80.65)	120,916 (82.47)	122,803 (87.65)	112,375 (87.93)	101,869 (86.81)	48,969 (89.12)	47,892 (87.32)	44,497 (85.82)
Yes	137,537 (14.99)	43,344 (19.35)	25,705 (17.53)	17,300 (12.35)	15,429 (12.07)	15,474 (13.19)	5979 (10.88)	6956 (12.68)	7350 (14.18)
Suicidal attempts, n (%)									
No	886,696 (96.65)	213,514 (95.34)	140,582 (95.88)	136,337 (97.31)	124,640 (97.52)	113,739 (96.93)	53,827 (97.96)	53,603 (97.73)	50,454 (97.31)
Yes	30,765 (3.35)	10,433 (4.66)	6039 (4.12)	3766 (2.69)	3164 (2.48)	3604 (3.07)	1121 (2.04)	1245 (2.27)	1393 (2.69)

*BMI* body mass index, *KYRBS* Korea Youth Risk Behavior Web-based Survey.

^a^According to Asia–Pacific guidelines, BMI is divided into 4 groups: underweight (< 18.5 kg/m^2^), normal (18.5–22.9 kg/m^2^), overweight (23.0–24.9 kg/m^2^), and obese (≥ 25.0 kg/m^2^).

^b^Stress was defined by receipt of mental health counseling owing to stress.

**Figure 2:**
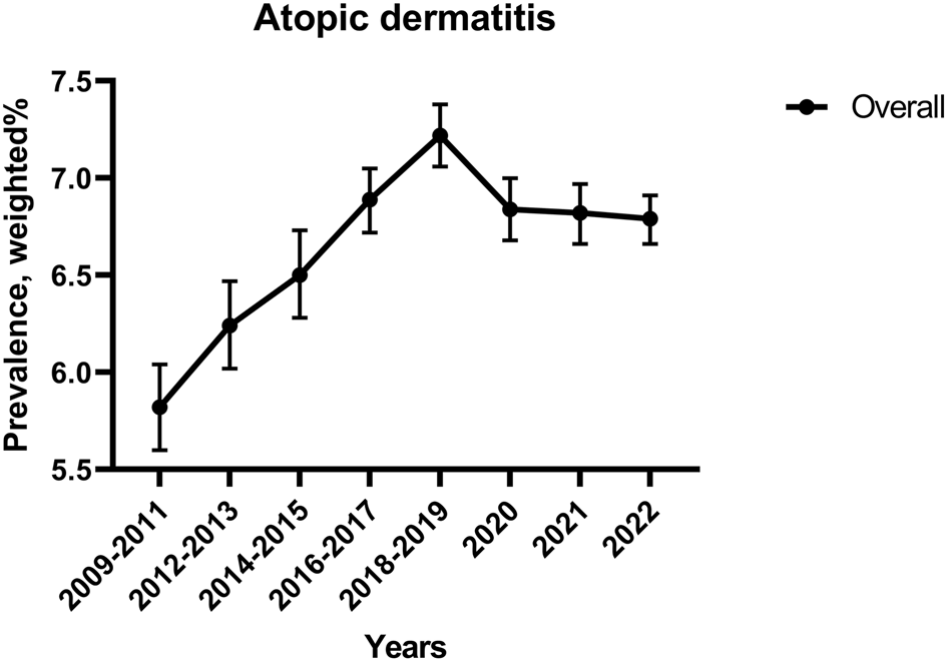
Longitudinal trend and prevalence of AD in Korean adolescents, 2009–2022. *AD* atopic dermatitis.

Table 2 and Fig. 2 shows the weighted prevalence of AD from 2009 to 2022, further stratified against socioeconomic factors. The weighted prevalence of AD increased from 6.79% (95% CI 6.66–6.91) in 2009–2011 to 7.22% (95% CI 7.06–7.38) in 2016–2017. However, there was a slight decrease immediately preceding the pandemic, from 2018–2019 (6.89%, 95% CI 6.72–7.05) to 2022 (5.82%, 95% CI 5.60–6.04). There was no significant difference between the pre-pandemic and pandemic in prevalence (β_diff_ − 0.041, 95% CI − 0.124 to 0.042) showing that the prevalence decreased since the start of the pandemic. Table 3 was analyzed to more closely examine the changes in AD prevalence during the pandemic period. The prevalence consistently showed a decreasing trend. Notably, from 2021 to 2022, there was a significant decrease (weighted OR 0.93, 95% CI 0.88 to 0.98). Table 4 shows the odds ratios of the socioeconomic factors of AD. Overall, from 2009 to 2022, high school grade, female sex, urban, high BMI group, lower-middle school performance, university graduated or higher parent’s highest educational level, middle household income, current alcohol consumption, smoker, severe stress, sadness and despair, suicidal thoughts, and suicidal attempts are observed as risk factor of AD. In addition, we calculated the ratio of OR for the pandemic/pre-pandemic period to analyze vulnerable groups during the pandemic. AD vulnerable groups during the pandemic were followed by; high school grade (ratio of OR 95% CI 1.07 [1.01 to 1.12]), middle household income (ratio of OR 95% CI 1.18 [1.09 to 1.27]), ≥ 10 days alcohol consumption (ratio of OR, 95% CI 1.25 [1.05 to 1.49]), and current smoker (ratio of OR 95% CI 1.10 [1.02 to 1.19]).

**Table 2:** The national trend of the prevalence of AD and β-coefficients and odds ratios before and during COVID-19 pandemic, weighted % (95% CI), in the KYRBS.

Characteristics	Pre-pandemic (2009–2019)					Pandemic (2020–2022)			Trend of the pre-pandemic era, β (95% CI)	Trend of the pandemic era, β (95% CI)	Trend difference, β_diff_ (95% CI)
Year	2009–2011	2012–2013	2014–2015	2016–2017	2018–2019	2020	2021	2022			
Overall	6.79 (6.66–6.91)	6.82 (6.66–6.97)	6.84 (6.68–7.00)	7.22 (7.06–7.38)	6.89 (6.72–7.05)	6.50 (6.28–6.73)	6.24 (6.02–6.47)	5.82 (5.60–6.04)	**0.006 (0.035 to 0.002)**	− **0.034 (**− **0.158 to 0.004)**	− 0.041 (− 0.124 to 0.042)
Grade, weighted (95% CI)											
7th–9th grade (middle school)	6.67 (6.50–6.84)	6.80 (6.59–7.02)	6.78 (6.57–6.99)	7.10 (6.88–7.32)	6.62 (6.39–6.85)	6.28 (5.95–6.61)	5.83 (5.55–6.12)	5.57 (5.27–5.88)	0.003 (0.016 to 0.003)	− **0.036 (**− **0.168 to 0.006)**	− 0.039 (− 0.126 to 0.049)
10th–12th grade (high school)	6.91 (6.71–7.10)	6.83 (6.62–7.04)	6.90 (6.66–7.13)	7.32 (7.09–7.56)	7.12 (6.88–7.36)	6.72 (6.41–7.03)	6.67 (6.32–7.01)	6.09 (5.77–6.41)	**0.009 (0.051 to 0.003)**	− **0.031 (**− **0.142 to 0.006)**	− 0.041 (− 0.119 to 0.037)
Sex, weighted (95% CI)											
Male	5.88 (5.71–6.04)	5.76 (5.57–5.94)	5.81 (5.61–6.01)	6.09 (5.89–6.29)	5.80 (5.59–6.01)	5.51 (5.22–5.80)	5.35 (5.06–5.65)	5.00 (4.72–5.28)	0.002 (0.012 to 0.003)	− **0.026 (**− **0.127 to 0.006)**	− 0.028 (− 0.094 to 0.039)
Female	7.80 (7.61–8.00)	7.98 (7.75–8.21)	7.97 (7.74–8.21)	8.46 (8.21–8.70)	8.06 (7.81–8.31)	7.57 (7.24–7.89)	7.19 (6.85–7.53)	6.70 (6.36–7.03)	**0.010 (0.054 to 0.004)**	− **0.045 (**− **0.191 to 0.007)**	− 0.055 (− 0.157 to 0.047)
Region of residence, weighted (95% CI)											
Rural	6.68 (6.50–6.85)	6.74 (6.52–6.95)	6.76 (6.51–7.01)	7.15 (6.92–7.37)	6.65 (6.42–6.89)	6.48 (6.14–6.82)	6.22 (5.89–6.55)	6.09 (5.75–6.43)	0.004 (0.025 to 0.003)	− **0.019 (**− **0.089 to 0.007)**	− 0.024 (− 0.073 to 0.025)
Urban	6.89 (6.70–7.08)	6.88 (6.67–7.09)	6.91 (6.70–7.11)	7.28 (7.05–7.51)	7.06 (6.83–7.29)	6.52 (6.22–6.82)	6.26 (5.96–6.56)	5.63 (5.34–5.92)	**0.008 (0.041 to 0.003)**	− **0.045 (**− **0.209 to 0.006)**	− 0.053 (− 0.162 to 0.056)
BMI group, weighted (95% CI)^a^											
Underweight	6.27 (6.04–6.50)	5.71 (5.44–5.97)	6.09 (5.82–6.36)	6.25 (5.93–6.57)	5.99 (5.66–6.32)	5.92 (5.42–6.42)	5.52 (5.09–5.96)	5.35 (4.91–5.79)	− 0.001 (− 0.004 to 0.005)	− **0.023 (**− **0.113 to 0.009)**	− 0.022 (− 0.083 to 0.039)
Normal	6.71 (6.54–6.88)	6.82 (6.62–7.03)	6.87 (6.66–7.08)	7.14 (6.91–7.37)	6.78 (6.54–7.01)	6.54 (6.22–6.86)	6.37 (6.04–6.69)	5.59 (5.27–5.91)	0.005 (0.029 to 0.003)	− **0.037 (**− **0.170 to 0.006)**	− 0.042 (− 0.132 to 0.047)
Overweight	7.27 (6.85–7.69)	7.92 (7.44–8.40)	7.43 (6.96–7.91)	8.24 (7.77–8.71)	7.47 (7.02–7.92)	6.67 (6.06–7.28)	6.22 (5.60–6.84)	6.21 (5.57–6.85)	0.008 (0.042 to 0.007)	− **0.043 (**− **0.190 to 0.013)**	− 0.051 (− 0.154 to 0.052)
Obese	7.75 (7.32–8.19)	7.96 (7.44–8.48)	7.40 (6.92–7.88)	7.82 (7.37–8.27)	7.59 (7.15–8.03)	6.97 (6.39–7.55)	6.57 (5.99–7.15)	6.02 (5.48–6.57)	− 0.005 (− 0.024 to 0.007)	− **0.051 (**− **0.223 to 0.012)**	− 0.046 (− 0.164 to 0.072)
Unknown	8.25 (7.48–9.02)	8.60 (7.65–9.54)	8.19 (7.28–9.10)	8.93 (7.94–9.92)	9.23 (8.11–10.35)	6.63 (5.12–8.14)	7.84 (6.28–9.40)	11.25 (9.32–13.18)	0.022 (0.109 to 0.015)	0.066 (0.265 to 0.035)	0.044 (− 0.080 to 0.168)
School performance, weighted (95% CI)											
Low (0–19 percentile)	7.19 (6.83–7.54)	7.35 (6.94–7.76)	7.13 (6.65–7.61)	8.08 (7.57–8.60)	7.71 (7.17–8.25)	6.74 (6.05–7.44)	6.76 (6.07–7.45)	6.98 (6.21–7.74)	**0.017 (0.092 to 0.007)**	− 0.023 (− 0.099 to 0.015)	− 0.040 (− 0.111 to 0.031)
Lower-middle (20–39 percentile)	6.92 (6.67–7.17)	7.02 (6.73–7.31)	6.89 (6.60–7.19)	7.57 (7.24–7.91)	7.11 (6.76–7.45)	6.53 (6.06–6.99)	6.29 (5.79–6.79)	5.45 (4.99–5.91)	**0.010 (0.052 to 0.005)**	− **0.052 (**− **0.238 to 0.009)**	− 0.062 (− 0.187 to 0.064)
Middle (40–59 percentile)	6.48 (6.24–6.73)	6.46 (6.18–6.74)	6.73 (6.46–7.00)	6.90 (6.62–7.17)	6.61 (6.31–6.91)	6.48 (6.06–6.91)	6.13 (5.70–6.56)	5.59 (5.19–5.99)	0.007 (0.041 to 0.004)	− **0.034 (**− **0.157 to 0.008)**	− 0.041 (− 0.126 to 0.043)
Upper-middle (60–79 percentile)	6.74 (6.48–6.99)	6.61 (6.31–6.91)	6.76 (6.48–7.05)	7.02 (6.72–7.31)	6.92 (6.61–7.24)	6.33 (5.87–6.79)	6.17 (5.74–6.60)	5.85 (5.41–6.30)	0.008 (0.043 to 0.005)	− **0.034 (**− **0.157 to 0.009)**	− 0.042 (− 0.127 to 0.043)
High (80–100 percentile)	6.87 (6.49–7.25)	7.07 (6.61–7.52)	6.90 (6.47–7.32)	7.07 (6.64–7.50)	6.44 (6.03–6.86)	6.65 (5.99–7.31)	6.16 (5.52–6.80)	6.07 (5.47–6.66)	− 0.008 (− 0.042 to 0.007)	− 0.016 (− 0.073 to 0.012)	− 0.008 (− 0.057 to 0.041)
Parent’s highest educational level, weighted (95% CI)											
Middle school graduated or under	5.69 (5.34–6.04)	5.76 (5.40–6.13)	6.07 (5.71–6.44)	6.21 (5.83–6.58)	6.03 (5.75–6.30)	5.63 (5.28–5.98)	5.47 (5.10–5.83)	5.75 (5.37–6.13)	0.010 (0.059 to 0.005)	− 0.010 (− 0.048 to 0.008)	− 0.020 (− 0.058 to 0.019)
High school graduated	6.50 (5.83–7.18)	6.53 (5.61–7.44)	7.00 (5.84–8.17)	7.70 (6.10–9.30)	8.54 (6.43–10.65)	6.68 (3.78–9.57)	7.39 (4.12–10.67)	9.11 (5.59–12.62)	**0.043 (0.214 to 0.020)**	0.016 (0.069 to 0.065)	− 0.027 (− 0.124 to 0.070)
University graduated or higher	6.72 (6.51–6.94)	6.94 (6.68–7.19)	7.01 (6.73–7.29)	7.58 (7.25–7.91)	7.29 (6.89–7.69)	7.11 (6.52–7.70)	6.96 (6.38–7.55)	5.57 (4.99–6.15)	**0.019 (0.100 to 0.005)**	− **0.050 (**− **0.224 to 0.011)**	− 0.069 (− 0.196 to 0.057)
Unknown	7.09 (6.91–7.27)	6.99 (6.78–7.20)	6.94 (6.73–7.16)	7.30 (7.10–7.51)	7.19 (6.95–7.43)	6.90 (6.57–7.23)	6.56 (6.26–6.87)	5.89 (5.59–6.18)	0.005 (0.027 to 0.003)	− **0.042 (**− **0.194 to 0.006)**	− 0.048 (− 0.148 to 0.053)
Household income, weighted (95% CI)											
Middle lower or lower	7.36 (7.09–7.64)	7.72 (7.40–8.04)	7.84 (7.47–8.21)	8.41 (7.98–8.84)	8.29 (7.79–8.78)	8.15 (7.41–8.88)	8.37 (7.57–9.18)	7.65 (6.97–8.34)	**0.026 (0.133 to 0.006)**	− 0.016 (− 0.065 to 0.014)	− 0.042 (− 0.117 to 0.032)
Middle	6.57 (6.37–6.76)	6.59 (6.38–6.80)	6.72 (6.49–6.94)	7.25 (7.01–7.49)	6.69 (6.47–6.92)	6.12 (5.82–6.43)	6.23 (5.92–6.53)	5.53 (5.21–5.86)	**0.010 (0.055 to 0.003)**	− **0.034 (**− **0.156 to 0.006)**	− 0.043 (− 0.128 to 0.042)
Middle higher or higher	6.68 (6.44–6.91)	6.54 (6.28–6.79)	6.52 (6.27–6.76)	6.74 (6.49–6.99)	6.66 (6.41–6.91)	6.43 (6.06–6.79)	5.68 (5.35–6.02)	5.67 (5.34–6.01)	0.002 (0.009 to 0.004)	− **0.037 (**− **0.174 to 0.007)**	− 0.038 (− 0.129 to 0.052)
Alcohol consumption, weighted (95% CI)											
Non-drinker	6.58 (6.44–6.73)	6.66 (6.50–6.83)	6.71 (6.54–6.87)	7.11 (6.94–7.29)	6.66 (6.48–6.84)	6.36 (6.12–6.60)	6.10 (5.87–6.33)	5.67 (5.44–5.89)	**0.007 (0.037 to 0.003)**	− **0.032 (**− **0.149 to 0.005)**	− 0.039 (− 0.118 to 0.040)
1–2 day	7.10 (6.73–7.47)	7.17 (6.71–7.62)	7.20 (6.72–7.69)	7.43 (6.91–7.95)	7.72 (7.18–8.26)	7.48 (6.56–8.40)	6.26 (5.41–7.12)	6.23 (5.37–7.09)	0.014 (0.076 to 0.007)	− **0.055 (**− **0.258 to 0.016)**	− 0.070 (− 0.211 to 0.072)
3–5 day	7.59 (6.94–8.23)	7.82 (6.99–8.65)	7.23 (6.31–8.15)	8.35 (7.39–9.31)	7.49 (6.56–8.42)	6.88 (5.24–8.52)	8.45 (6.52–10.38)	7.22 (5.54–8.91)	0.004 (0.019 to 0.013)	0.005 (0.022 to 0.031)	0.001 (− 0.004 to 0.007)
6–9 day	7.67 (6.85–8.49)	7.50 (6.49–8.51)	7.86 (6.35–9.36)	6.48 (5.23–7.74)	8.02 (6.60–9.43)	7.69 (5.46–9.92)	8.14 (5.28–11.00)	6.82 (4.71–8.93)	− 0.004 (− 0.021 to 0.018)	− 0.032 (− 0.140 to 0.042)	− 0.028 (− 0.121 to 0.065)
≥ 10 day	9.20 (8.38–10.02)	8.71 (7.67–9.75)	9.23 (8.01–10.46)	10.27 (8.78–11.76)	10.65 (9.28–12.01)	9.82 (7.31–12.34)	11.24 (8.48–14.01)	10.06 (7.36–12.76)	0.039 (0.189 to 0.018)	− 0.006 (− 0.023 to 0.048)	− 0.045 (− 0.138 to 0.048)
Smoking status, weighted (95% CI)											
Non-smoker	6.70 (6.55–6.86)	6.72 (6.54–6.89)	6.85 (6.68–7.03)	7.19 (7.02–7.36)	6.68 (6.50–6.85)	6.45 (6.21–6.68)	6.13 (5.90–6.36)	5.71 (5.48–5.94)	0.005 (0.026 to 0.003)	− **0.032 (**− **0.149 to 0.005)**	− 0.037 (− 0.115 to 0.041)
Smoker	7.01 (6.77–7.26)	7.14 (6.83–7.46)	6.80 (6.45–7.15)	7.43 (7.01–7.85)	8.19 (7.72–8.67)	6.98 (6.24–7.72)	7.26 (6.46–8.06)	7.00 (6.17–7.84)	**0.021 (0.111 to 0.006)**	− **0.037 (**− **0.160 to 0.015)**	− 0.058 (− 0.160 to 0.044)
Stress status, weighted (95% CI)^b^											
None	5.42 (4.68–6.16)	5.60 (4.87–6.33)	5.52 (4.82–6.21)	6.00 (5.24–6.76)	5.05 (4.33–5.78)	4.53 (3.57–5.49)	5.13 (3.99–6.27)	4.62 (3.48–5.77)	− 0.003 (− 0.017 to 0.012)	− 0.007 (− 0.035 to 0.022)	− 0.004 (− 0.035 to 0.028)
Mild	5.06 (4.78–5.35)	5.34 (5.00–5.69)	5.24 (4.93–5.55)	5.35 (5.03–5.68)	5.08 (4.72–5.44)	5.18 (4.70–5.67)	4.47 (4.01–4.93)	4.63 (4.09–5.16)	0.001 (0.007 to 0.005)	− 0.021 (− 0.108 to 0.010)	− 0.022 (− 0.082 to 0.037)
Moderate	6.27 (6.09–6.46)	6.28 (6.06–6.50)	6.41 (6.19–6.62)	6.81 (6.57–7.05)	6.27 (6.01–6.52)	6.25 (5.92–6.58)	5.78 (5.45–6.11)	5.13 (4.80–5.46)	0.006 (0.035 to 0.004)	− **0.039 (**− **0.182 to 0.007)**	− 0.045 (− 0.140 to 0.051)
High	7.43 (7.19–7.66)	7.40 (7.12–7.68)	8.02 (7.70–8.33)	8.17 (7.86–8.49)	7.76 (7.44–8.08)	7.39 (6.94–7.83)	7.27 (6.82–7.73)	6.44 (6.03–6.84)	**0.016 (0.082 to 0.005)**	− **0.041 (**− **0.180 to 0.008)**	− 0.056 (− 0.158 to 0.045)
Severe	9.01 (8.61–9.41)	9.30 (8.83–9.77)	8.85 (8.32–9.38)	9.86 (9.27–10.45)	9.83 (9.29–10.37)	8.77 (7.90–9.65)	8.25 (7.42–9.08)	8.35 (7.59–9.11)	**0.021 (0.104 to 0.008)**	− **0.050 (**− **0.208 to 0.015)**	− 0.071 (− 0.193 to 0.050)
Sadness and despair, weighted (95% CI)											
No	6.00 (5.84–6.15)	6.13 (5.96–6.31)	6.19 (6.03–6.36)	6.58 (6.41–6.76)	6.18 (6.00–6.36)	6.00 (5.74–6.25)	5.56 (5.32–5.80)	5.35 (5.10–5.60)	**0.009 (0.051 to 0.003)**	− **0.029 (**− **0.139 to 0.005)**	− 0.038 (− 0.114 to 0.038)
Yes	8.19 (7.98–8.41)	8.36 (8.07–8.65)	8.77 (8.43–9.10)	9.12 (8.76–9.47)	8.73 (8.40–9.06)	8.00 (7.52–8.47)	8.10 (7.63–8.58)	6.98 (6.55–7.42)	**0.019 (0.099 to 0.005)**	− **0.052 (**− **0.218 to 0.009)**	− 0.071 (− 0.194 to 0.052)
Suicidal thoughts, weighted (95% CI)											
No	6.37 (6.23–6.51)	6.43 (6.27–6.59)	6.57 (6.41–6.73)	6.86 (6.69–7.03)	6.48 (6.31–6.66)	6.29 (6.05–6.53)	5.84 (5.61–6.07)	5.49 (5.26–5.73)	**0.007 (0.040 to 0.003)**	− **0.034 (**− **0.160 to 0.005)**	− 0.041 (− 0.126 to 0.043)
Yes	8.52 (8.20–8.84)	8.65 (8.27–9.03)	8.77 (8.29–9.25)	9.85 (9.33–10.37)	9.54 (9.04–10.03)	8.26 (7.44–9.07)	8.99 (8.28–9.70)	7.81 (7.13–8.49)	**0.031 (0.155 to 0.007)**	− **0.046 (**− **0.191 to 0.014)**	− 0.078 (− 0.204 to 0.048)
Suicidal attempts, weighted (95% CI)											
No	6.63 (6.50–6.76)	6.68 (6.53–6.83)	6.74 (6.58–6.90)	7.12 (6.96–7.29)	6.77 (6.61–6.94)	6.46 (6.23–6.68)	6.15 (5.93–6.37)	5.67 (5.45–5.89)	**0.008 (0.043 to 0.002)**	− **0.036 (**− **0.167 to 0.005)**	− 0.044 (− 0.132 to 0.044)
Yes	10.06 (9.34–10.78)	9.97 (9.14–10.80)	10.60 (9.55–11.66)	11.15 (9.92–12.38)	10.49 (9.45–11.53)	8.70 (6.74–10.65)	10.32 (8.56–12.08)	11.55 (9.65–13.45)	0.021 (0.096 to 0.015)	0.041 (0.157 to 0.035)	0.020 (− 0.054 to 0.093)

*AD* atopic dermatitis, *BMI* body mass index, *CI* confidence interval, *OR* odds ratio.

^a^According to Asia–Pacific guidelines, BMI is divided into 4 groups: underweight (< 18.5 kg/m^2^), normal (18.5–22.9 kg/m^2^), overweight (23.0–24.9 kg/m^2^), and obese (≥ 25.0 kg/m^2^).

^b^Stress was defined by receipt of mental health counseling owing to stress.

Significant values are in bold (*p* < 0.05).

**Table 3:** Comparison with the previous year by the wORs for prevalence of AD and each socioeconomic factor, weighted % (95% CI).

Characteristics	2020 versus 2019 (reference)	2021 versus 2020 (reference)	2022 versus 2021 (reference)
	wOR (95% CI)	wOR (95% CI)	wOR (95% CI)
Overall	0.99 (0.94 to 1.05)	0.96 (0.91 to 1.01)	**0.93 (0.88 to 0.98)**
Grade, weighted (95% CI)			
7th–9th grade (middle school)	1.00 (0.93 to 1.08)	**0.93 (0.86 to 1.00)**	0.95 (0.88 to 1.03)
10th–12th grade (high school)	0.98 (0.92 to 1.06)	0.99 (0.92 to 1.07)	**0.91 (0.84 to 0.98)**
Sex, weighted (95% CI)			
Male	0.99 (0.91 to 1.07)	0.97 (0.90 to 1.05)	0.93 (0.86 to 1.01)
Female	0.99 (0.93 to 1.06)	0.95 (0.88 to 1.01)	**0.93 (0.86 to 1.00)**
Region of residence, weighted (95% CI)			
Rural	1.01 (0.94 to 1.10)	0.96 (0.88 to 1.04)	0.98 (0.90 to 1.06)
Urban	0.97 (0.91 to 1.05)	0.96 (0.89 to 1.03)	**0.89 (0.83 to 0.96)**
BMI group, weighted (95% CI)^a^			
Underweight	1.06 (0.93 to 1.20)	0.93 (0.82 to 1.05)	0.97 (0.86 to 1.09)
Normal	1.02 (0.95 to 1.11)	0.97 (0.90 to 1.05)	**0.87 (0.80 to 0.95)**
Overweight	0.93 (0.81 to 1.07)	0.93 (0.80 to 1.07)	1.00 (0.86 to 1.16)
Obese	0.97 (0.85 to 1.10)	0.94 (0.83 to 1.07)	0.91 (0.80 to 1.04)
Unknown	**0.59 (0.44 to 0.80)**	1.20 (0.87 to 1.66)	**1.49 (1.12 to 1.99)**
School performance, weighted (95% CI)			
Low (0–19 percentile)	0.90 (0.77 to 1.05)	1.00 (0.86 to 1.17)	1.03 (0.88 to 1.21)
Lower-middle (20–39 percentile)	0.94 (0.85 to 1.05)	0.96 (0.86 to 1.08)	**0.86 (0.76 to 0.97)**
Middle (40–59 percentile)	1.09 (0.99 to 1.21)	0.94 (0.85 to 1.04)	0.91 (0.82 to 1.01)
Upper-middle (60–79 percentile)	0.93 (0.83 to 1.03)	0.97 (0.87 to 1.09)	0.95 (0.85 to 1.06)
High (80–100 percentile)	1.07 (0.93 to 1.24)	0.92 (0.79 to 1.07)	0.98 (0.85 to 1.15)
Parent’s highest educational level, weighted (95% CI)			
Middle school graduated or under	0.93 (0.85 to 1.01)	0.97 (0.88 to 1.07)	1.06 (0.96 to 1.17)
High school graduated	0.64 (0.35 to 1.18)	1.12 (0.57 to 2.17)	1.26 (0.66 to 2.38)
University graduated or higher	1.03 (0.90 to 1.17)	0.98 (0.86 to 1.11)	**0.79 (0.68 to 0.91)**
Unknown	1.00 (0.92 to 1.08)	0.95 (0.88 to 1.02)	**0.89 (0.83 to 0.96)**
Household income, weighted (95% CI)			
Middle lower or lower	1.01 (0.88 to 1.15)	1.03 (0.89 to 1.19)	0.91 (0.79 to 1.05)
Middle	0.98 (0.91 to 1.05)	1.02 (0.95 to 1.10)	**0.88 (0.81 to 0.96)**
Middle higher or higher	1.00 (0.92 to 1.09)	**0.88 (0.80 to 0.96)**	1.00 (0.91 to 1.09)
Alcohol consumption, weighted (95% CI)			
Non-drinker	1.01 (0.96 to 1.07)	0.96 (0.90 to 1.01)	**0.93 (0.87 to 0.98)**
1–2 day	0.98 (0.83 to 1.17)	0.83 (0.68 to 1.01)	0.99 (0.81 to 1.22)
3–5 day	0.89 (0.64 to 1.23)	1.25 (0.87 to 1.79)	0.84 (0.59 to 1.20)
6–9 day	1.09 (0.71 to 1.66)	1.06 (0.65 to 1.74)	0.83 (0.50 to 1.37)
≥ 10 day	0.80 (0.56 to 1.14)	1.16 (0.78 to 1.73)	0.88 (0.59 to 1.33)
Smoking status, weighted (95% CI)			
Non-smoker	1.02 (0.96 to 1.08)	0.95 (0.90 to 1.00)	0.93 (0.87 to 0.98)
Smoker	**0.85 (0.73 to 0.99)**	1.04 (0.89 to 1.23)	0.96 (0.81 to 1.15)
Stress status, weighted (95% CI)^b^			
None	0.84 (0.62 to 1.14)	1.14 (0.83 to 1.57)	0.90 (0.63 to 1.27)
Mild	1.13 (0.98 to 1.31)	**0.86 (0.74 to 0.99)**	1.04 (0.88 to 1.22)
Moderate	1.06 (0.97 to 1.15)	0.92 (0.85 to 1.00)	**0.88 (0.81 to 0.97)**
High	1.00 (0.91 to 1.10)	0.98 (0.90 to 1.08)	**0.88 (0.80 to 0.97)**
Severe	0.90 (0.78 to 1.03)	0.94 (0.80 to 1.09)	1.01 (0.87 to 1.18)
Sadness and despair, weighted (95% CI)			
No	1.02 (0.96 to 1.09)	**0.92 (0.87 to 0.98)**	0.96 (0.90 to 1.03)
Yes	0.96 (0.88 to 1.05)	1.01 (0.93 to 1.11)	**0.85 (0.78 to 0.93)**
Suicidal thoughts, weighted (95% CI)			
No	1.04 (0.98 to 1.10)	**0.93 (0.87 to 0.98)**	**0.94 (0.88 to 1.00)**
Yes	**0.84 (0.73 to 0.96)**	1.10 (0.96 to 1.26)	**0.86 (0.75 to 0.98)**
Suicidal attempts, weighted (95% CI)			
No	1.00 (0.95 to 1.06)	0.95 (0.90 to 1.00)	0.92 (0.87 to 0.97)
Yes	0.80 (0.59 to 1.07)	1.21 (0.89 to 1.65)	1.13 (0.87 to 1.48)

Significant values are in bold (*p* < 0.05).

**Table 4:** Ratio of ORs for association between prevalence of AD and each socioeconomic factor, 2009–2022.

	Overall OR	Pre-pandemic era OR	Pandemic era OR	Ratio of ORs
	2009–2022; weighted OR	2009–2019; weighted OR	2020–2022; weighted OR	(Pandemic/pre-pandemic); weighted ratio of OR
Grade, weighted (95% CI)				
7th–9th grade (middle school)	1.00 (reference)	1.00 (reference)	1.00 (reference)	1.00 (reference)
10th–12th grade (high school)	**1.05 (1.03 to 1.07)**	**1.04 (1.01 to 1.06)**	**1.11 (1.06 to 1.16)**	**1.07 (1.01 to 1.12)**
Sex, weighted (95% CI)				
Male	1.00 (reference)	1.00 (reference)	1.00 (reference)	1.00 (reference)
Female	**1.40 (1.37 to 1.42)**	**1.40 (1.37 to 1.43)**	**1.38 (1.32 to 1.44)**	0.99 (0.94 to 1.04)
Region of residence, weighted (95% CI)				
Rural	1.00 (reference)	1.00 (reference)	1.00 (reference)	1.00 (reference)
Urban	**1.02 (1.00 to 1.04)**	**1.03 (1.01 to 1.06)**	0.98 (0.94 to 1.02)	**0.95 (0.91 to 1.00)**
BMI group, weighted (95% CI)^a^				
Underweight	1.00 (reference)	1.00 (reference)	1.00 (reference)	1.00 (reference)
Normal	**1.13 (1.10 to 1.16)**	**1.13 (1.11 to 1.16)**	**1.11 (1.05 to 1.18)**	0.98 (0.92 to 1.05)
Overweight	**1.25 (1.21 to 1.29)**	**1.28 (1.23 to 1.32)**	**1.15 (1.06 to 1.25)**	**0.90 (0.82 to 0.98)**
Obese	**1.25 (1.21 to 1.30)**	**1.29 (1.24 to 1.34)**	**1.18 (1.10 to 1.27)**	**0.92 (0.84 to 0.99)**
School performance, weighted (95% CI)				
Low (0–19 percentile)	1.00 (reference)	1.00 (reference)	1.00 (reference)	1.00 (reference)
Lower-middle (20–39 percentile)	**1.13 (1.10 to 1.17)**	**1.13 (1.09 to 1.17)**	**1.13 (1.05 to 1.23)**	1.00 (0.92 to 1.09)
Middle (40–59 percentile)	**1.07 (1.04 to 1.09)**	**1.07 (1.04 to 1.10)**	1.01 (0.94 to 1.07)	0.94 (0.88 to 1.01)
Upper-middle (60–79 percentile)	**1.03 (1.00 to 1.05)**	**1.03 (1.00 to 1.06)**	1.01 (0.95 to 1.07)	0.98 (0.92 to 1.05)
High (80–100 percentile)	**1.04 (1.01 to 1.08)**	**1.04 (1.01 to 1.08)**	1.04 (0.96 to 1.12)	1.00 (0.92 to 1.09)
Parent’s highest educational level, weighted (95% CI)				
Middle school graduated or under	1.00 (reference)	1.00 (reference)	1.00 (reference)	1.00 (reference)
High school graduated	**1.19 (1.10 to 1.28)**	**0.87 (0.80 to 0.94)**	**0.71 (0.55 to 0.93)**	0.82 (0.62 to 1.07)
University graduated or higher	**1.21 (1.17 to 1.24)**	**1.03 (0.96 to 1.11)**	**0.84 (0.65 to 1.10)**	0.82 (0.62 to 1.07)
Household income, weighted (95% CI)				
Middle lower or lower	1.00 (reference)	1.00 (reference)	1.00 (reference)	1.00 (reference)
Middle	**1.22 (1.19 to 1.25)**	**1.18 (1.15 to 1.22)**	**1.39 (1.30 to 1.49)**	**1.18 (1.09 to 1.27)**
Middle higher or higher	1.02 (1.00 to 1.04)	1.02 (0.99 to 1.04)	1.01 (0.96 to 1.06)	0.99 (0.94 to 1.05)
Alcohol consumption, weighted (95% CI)				
Non-drinker	1.00 (reference)	1.00 (reference)	1.00 (reference)	1.00 (reference)
1–2 day	**1.10 (1.06 to 1.13)**	**1.09 (1.05 to 1.12)**	**1.10 (1.01 to 1.20)**	1.01 (0.92 to 1.11)
3–5 day	**1.17 (1.12 to 1.23)**	**1.15 (1.09 to 1.22)**	**1.26 (1.09 to 1.46)**	1.10 (0.94 to 1.28)
6–9 day	**1.16 (1.08 to 1.24)**	**1.14 (1.05 to 1.22)**	**1.26 (1.03 to 1.54)**	1.11 (0.89 to 1.37)
≥ 10 day	**1.49 (1.41 to 1.58)**	**1.44 (1.36 to 1.53)**	**1.80 (1.52 to 2.12)**	**1.25 (1.05 to 1.49)**
Smoking status, weighted (95% CI)				
Non-smoker	1.00 (reference)	1.00 (reference)	1.00 (reference)	1.00 (reference)
Smoker	**1.08 (1.05 to 1.10)**	**1.06 (1.03 to 1.08)**	**1.17 (1.09 to 1.26)**	**1.10 (1.02 to 1.19)**
Stress status, weighted (95% CI)^b^				
None	1.00 (reference)	1.00 (reference)	1.00 (reference)	1.00 (reference)
Mild	0.95 (0.89 to 1.01)	0.94 (0.88 to 1.01)	1.01 (0.87 to 1.17)	1.07 (0.91 to 1.27)
Moderate	**1.18 (1.11 to 1.25)**	**1.17 (1.09 to 1.25)**	**1.22 (1.06 to 1.40)**	1.04 (0.89 to 1.22)
High	**1.44 (1.36 to 1.53)**	**1.42 (1.33 to 1.52)**	**1.51 (1.31 to 1.74)**	1.06 (0.91 to 1.24)
Severe	**1.77 (1.66 to 1.88)**	**1.75 (1.64 to 1.88)**	**1.84 (1.59 to 2.14)**	1.05 (0.89 to 1.24)
Sadness and despair, weighted (95% CI)				
No	1.00 (reference)	1.00 (reference)	1.00 (reference)	1.00 (reference)
Yes	**1.41 (1.38 to 1.44)**	**1.41 (1.38 to 1.44)**	**1.39 (1.33 to 1.46)**	0.99 (0.94 to 1.04)
Suicidal thoughts, weighted (95% CI)				
No	1.00 (reference)	1.00 (reference)	1.00 (reference)	1.00 (reference)
Yes	**1.41 (1.38 to 1.44)**	**1.40 (1.36 to 1.43)**	**1.46 (1.37 to 1.55)**	1.04 (0.98 to 1.12)
Suicidal attempts, weighted (95% CI)				
No	1.00 (reference)	1.00 (reference)	1.00 (reference)	1.00 (reference)
Yes	**1.61 (1.54 to 1.68)**	**1.58 (1.51 to 1.66)**	**1.77 (1.57 to 2.00)**	1.12 (0.98 to 1.28)

*AD* atopic dermatitis, *BMI* body mass index, *CI* confidence interval, *OR* odds ratio.

^a^According to Asia–Pacific guidelines, BMI is divided into 4 groups: underweight (< 18.5 kg/m^2^), normal (18.5–22.9 kg/m^2^), overweight (23.0–24.9 kg/m^2^), and obese (≥ 25.0 kg/m^2^).

^b^Stress was defined by receipt of mental health counseling owing to stress.

Significant values are in bold (*p* < 0.05).

In Table S3, risk factors such as high school grade, female sex, high BMI group, low school performance, high school graduated parent’s highest educational level, current alcohol consumption, smoker, severe stress, sadness and despair, suicidal thoughts, and suicidal attempts were identified during the pandemic in 2020–2022, respectively.

## Discussion

### Key results

In this study we found the prevalence of AD to be 5.82% in 2022 among adolescents aged 13 to 18 years. Analyzing the prevalence of AD is important because this allergic disease has a significant impact on the quality of life, particularly among adolescents. The prevalence of AD continued to increase prior to the pandemic. However, the most significant decline in prevalence occurred after the COVID-19 pandemic began, with a smaller decline observed during the pandemic. Examining this prevalence contributes to understanding the scope of the COVID-19 pandemic and its influences on allergic disease trends. Additionally, current alcohol consumption, smoker, severe stress, sadness and despair, suicidal thoughts, and suicidal attempts are observed as risk factors of AD. These results could also provide a new perspective on coping with AD.

### Global epidemiology and mechanism

In South Korea, the first cases of COVID-19 were discovered in January 2020, and the severity of the pandemic increased over time^37^. One hypothesis for this phenomenon could be the reduced exposure to allergens due to the changes during COVID-19. Several significant social and political changes occurred during this period, which could have influenced the level of allergen exposure. Because the most drastic of measures included social distancing, lockdowns, closures of public places, decreased physical interaction, mask mandates, and other self-isolating actions^38–40^. Furthermore, during the COVID-19, there was a decrease in air pollution in South Korea^41^, which is consistent with decreased smoking and automobile traffic resulting from decreased socialization^42^. Air pollution has previously been linked to AD as air particulates can damage the skin barrier through oxidative stress, thus triggering immune dysregulation which can potentially increase inflammation of AD^43,44^. Heavy metals exposure (a factor of air pollution) has similarly been associated with allergic diseases, acting as a direct activator of Th2 cells as well as an adjuvant, increasing the effect of other allergens^42^. In addition, the use of face masks has further enhanced the protective effect as studies have shown that they reduce allergic diseases, particularly AD^45,46^. While face masks could have an inflammatory effect on facial dermatoses due to increased skin hydration, temperature, and sebum secretion, it has been found that in patients with AD, facial eczema is deceased due to covering sensitive areas^46^. In 2022, many COVID-19 regulations began to be rescinded, with South Korea removing the mask mandate and other social restrictions as the pandemic started to wind down. This return to pre-pandemic action could partially explain the decreased trend of AD during 2022^13^. However, although there is a decreasing trend, it is too early to determine the impact of reducing social restrictions in 2022, so future research is required.

In this study, we found that alcohol consumption and smoking are both associated with an increased risk of AD, which is consistent with the literature^9^. However, in Korean adolescents, we found a minor negative association between high BMI and AD, which is in contrasts with other sources, as overweight or obese individuals have been found to have an increased risk of AD through a small but important causal relationship^47^. Having suicidal thoughts was the only factor that demonstrated an increased risk of AD across the pandemic, 2020–2022. However, further research is necessary to fully understand the underlying mechanism behind this association. Falling rates of AD during COVID-19 may also be due to other factors. Firstly, the common allergens for AD have been found to be changing as patients are less sensitized to them and new allergens emerge^48^. Secondly, in the United States, it has been postulated that falling rates of allergic disease could be due to the changes in measurements^49^, but the KYRBS has remained consistent in its measurement and questions. Finally, a study in Colombia found that leading up to 2019, there was an increasing utilization of healthcare resources and healthcare visits, which correlated with an increased in the incidence and prevalence of AD^50^. The COVID-19 pandemic saw a decrease in healthcare utilization, including for allergic disease, as fewer people reported to hospitals during COVID-19 for fear of contracting SARS-CoV-2^51^. Furthermore, many patients with AD are treated by specialists, and data from other countries data outpatient dermatological visits during the COVID-19 pandemic, with AD being the most common reason for visits^52,53^. However, specific studies conducted in South Korea found no decrease in incidence in adolescent AD from 2019 to 2020, so changes in incidence may not fully explain the decreasing trend of AD during this period^54^.

### Strengths and limitations

We utilized the KYRBS, which serves as both the greatest strength and limitation of this study. The KYRBS is the only national database for the prevalence of allergic disease since the COVID-19 pandemic, with over one million Korean adolescents included in the study. The KYRBS is also integrated with the public school system in South Korea, as it is administered to most school-age adolescents via computers with anonymous online self-reporting^9^. However, this poses several limitations. First, the KYRBS excludes any student not in the Korean public education system, potentially excluding a segment of the adolescent population. Second, the KYRBS is a self-reporting questionnaire, which, although considered reliable based on a kappa coefficient of 0.78–0.80^55^, still yields subjective data that lacks objective measures. Third, in our study, we analyzed the data without considering the sensitivity and specificity of AD. Therefore, there is a limitation that the numbers may change when considering this. However, even if we consider this limitation, it is unlikely to significantly alter the overall trend of the results. Fourth, this study has limitations due to the lack of use of validated questionnaires, impacting the reliability and accuracy of its results. This common issue in self-report studies necessitates caution in interpreting data and may restrict the generalizability of the findings. Fifth, while the results of this study show an overall decreasing trend in the number of AD patients, this may be attributed to a reduction in doctor visits during the pandemic. However, our dataset lacks information on doctor visits, making this difficult to ascertain. Finally, the data is confined to one country, South Korea. Primary healthcare is highly valued worldwide^56^. However, contrary to this, according to statistics from the World Health Organization, many countries suffer from poor access to primary healthcare^57,58^. On the other hand, South Korea boasts excellent accessibility to primary healthcare^59^. This disparity may be attributed to cultural differences, the presence or absence of social insurance, among other factors, which could result in the trends in other countries being different.

## Conclusion

This study observed the prevalence of AD over a 14-year from 2009 to 2022, analyzing both the differences before and during the COVID-19 pandemic, as well as within the pandemic itself. The prevalence of AD increased before the pandemic, then decreased at the start of the pandemic and remained stable throughout the pandemic. This prevalence helps us understand the scope of the COVID-19 pandemic and its effects on allergic disease trends. In addition, risk factors of AD include alcohol consumption, smoking, severe stress, feelings of sadness and despair, suicidal ideation, and suicide attempts. Further research is necessary to understand whether these results are specific to the COVID-19 pandemic and whether the trend of AD will revert to pre-pandemic levels as the COVID-19 pandemic ends.

### Supplementary Information

Supplementary Tables.

## Data Availability

Data are available on reasonable request. Study protocol, statistical code: available from DKY (email: yonkkang@gmail.com). Data set: available from the Korea Disease Control and Prevention Agency (KDCA) through a data use agreement.
