# Supplementary material for: National prevalence of atopic dermatitis in Korean adolescents from 2009 to 2022
Source: Sci Rep. 2024 May 29;14:12391. doi: 10.1038/s41598-024-62475-4 (PMC11137070; doi:10.1038/s41598-024-62475-4)
Supplement: Supplementary file 1 — Supplementary Tables. [file 41598_2024_62475_MOESM1_ESM.docx]

| **Supplementary materials** |
| --- |

Original Article

**National prevalence of atopic dermatitis in Korean adolescents from 2009 to 2022**

**Running title:** Atopic dermatitis and South Korea

Mafaz Kattih,^1∥^ Hojae Lee,^2,3∥^ Hyesu Jo,^2,3∥^ Jinyoung Jeong,^2,4∥^ Hyejun Kim,^2,5∥^ Jaeyu Park,^2,3∥^ Hwi Yang,^2,3^ Ann Nguyen,^1^ Hyeon Jin Kim,^2,3^ Hyeri Lee,^2,3^ Minji Kim,^2,3^ Myeongcheol Lee,^2,3^ Rosie Kwon,^2,3^ Sunyoung Kim,^6^ Ai Koyanagi,^7^ Min Seo Kim,^8^ Masoud Rahmati,^9,10,11^ [Guillermo F. López Sánchez](https://streaklinks.com/BfMTWjzVFXnjyxjmNQoEXiV-/https%3A%2F%2Fpubmed.ncbi.nlm.nih.gov%2F%3Fsort%3Ddate%26size%3D200%26term%3DL%25C3%25B3pez%2BS%25C3%25A1nchez%2BGF%26cauthor_id%3D36808652?email=yonkkang%40gmail.com),^12^ Elena Dragioti,^13,14^ Ju Hee Kim,^15^ Selin Woo,^2^ Seong H. Cho,^16*^ Lee Smith,^17*^ Dong Keon Yon^2,3,4,15*^

^∥^ The authors contributed equally as co-first authors

***** The authors contributed equally as corresponding authors

***Corresponding authors**

**Seong H. Cho**, MD, PhD

Division of Allergy and Immunology, Department of Internal Medicine, USF Morsani College of Medicine, Tampa, FL, USA

Email: scho2@usf.edu

**Dong Keon Yon**, MD, PhD, FACAAI, FAAAAI

Department of Pediatrics, Kyung Hee University College of Medicine 23 Kyungheedae-ro, Dongdaemun-gu, Seoul 02447, South Korea

Tel: +82-2-6935-2476

Fax: +82-504-478-0201

Email: [yonkkang@gmail.com](mailto:yonkkang@gmail.com)

**Lee Smith**, PhD

Centre for Health, Performance and Wellbeing, Anglia Ruskin University, Cambridge CB1 1PT, UK

Email: Lee.Smith@aru.ac.uk

**Table S1.** Baseline characteristics of participating adolescents in KYRBS, weighted (95% CI)

| **Characteristics** | Total | Pre-pandemic (2009-2019) | | | | | During pandemic (2020-2022) | | |
| --- | --- | --- | --- | --- | --- | --- | --- | --- | --- |
| **Year** | 2009-2022 | 2009-2011 | 2012-2013 | 2014-2015 | 2016-2017 | 2018-2019 | 2020 | 2021 | 2022 |
| **Overall, n** | 917,461 | 223,947 | 146,621 | 140,103 | 127,804 | 117,343 | 54,948 | 54,848 | 51,847 |
| **Grade, weighted (95% CI)** | | | | | | | | | |
| 7th–9th grade (middle school) | 48.85 (48.41-49.28) | 50.07 (49.12-51.01) | 48.90 (47.80-50.00) | 47.65 (46.59-48.72) | 45.33 (44.26-46.41) | 47.13 (46.01-48.25) | 49.60 (48.00-51.19) | 50.96 (49.41-52.50) | 51.64 (49.98-53.31) |
| 10th–12th grade (high school) | 51.15 (50.72-51.59) | 49.93 (48.99-50.88) | 51.10 (50.00-52.20) | 52.35 (51.28-53.41) | 54.67 (53.59-55.74) | 52.87 (51.75-53.99) | 50.40 (48.81-52.00) | 49.04 (47.50-50.59) | 48.36 (46.70-50.02) |
| **Sex, weighted (95% CI)** | | | | | | | | | |
| Male | 52.13 (51.40-52.86) | 52.82 (51.15-54.49) | 52.40 (50.43-54.37) | 52.16 (50.22-54.10) | 52.12 (50.15-54.10) | 52.02 (50.13-53.90) | 51.86 (49.38-54.34) | 51.67 (49.28-54.05) | 51.56 (49.20-53.92) |
| Female | 47.87 (47.14-48.60) | 47.18 (45.51-48.85) | 47.60 (45.63-49.57) | 47.84 (45.90-49.78) | 47.88 (45.90-49.85) | 47.98 (46.10-49.87) | 48.14 (45.66-50.62) | 48.33 (45.95-50.72) | 48.44 (46.08-50.80) |
| **Region of residence, weighted (95% CI)** | | | | | | | | | |
| Urban | 43.76 (43.34-44.18) | 48.42 (47.49-49.35) | 44.25 (43.20-45.29) | 43.57 (42.55-44.58) | 43.21 (42.19-44.23) | 42.67 (41.63-43.71) | 42.23 (40.70-43.76) | 41.93 (40.43-43.42) | 41.54 (39.96-43.11) |
| Rural | 56.24 (55.82-56.66) | 51.59 (50.66-52.51) | 55.75 (54.71-56.80) | 56.43 (55.42-57.45) | 56.79 (55.77-57.81) | 57.33 (56.29-58.37) | 57.77 (56.24-59.30) | 58.08 (56.58-59.57) | 58.46 (56.89-60.04) |
| **BMI group, weighted (95% CI) ^a^** | | | | | | | | | |
| Underweight | 22.92 (22.77-23.06) | 26.00 (25.67-26.33) | 24.69 (24.34-25.04) | 23.58 (23.22-23.94) | 21.21 (20.85-21.56) | 20.81 (20.46-21.17) | 20.75 (20.23-21.28) | 21.08 (20.57-21.58) | 23.21 (22.65-23.78) |
| Normal | 50.14 (49.98-50.29) | 52.72 (52.41-53.02) | 52.83 (52.46-53.19) | 52.11 (51.72-52.49) | 50.91 (50.51-51.30) | 49.56 (49.16-49.95) | 47.37 (46.78-47.95) | 46.21 (45.65-46.76) | 46.26 (45.69-46.83) |
| Overweight | 11.59 (11.50-11.68) | 9.97 (9.80-10.14) | 10.60 (10.42-10.79) | 11.25 (11.05-11.46) | 12.08 (11.86-12.30) | 12.19 (11.97-12.41) | 13.14 (12.79-13.49) | 12.65 (12.29-13.00) | 11.97 (11.60-12.35) |
| Obese | 12.61 (12.48-12.75) | 8.09 (7.89-8.30) | 9.20 (8.96-9.44) | 10.14 (9.88-10.40) | 12.97 (12.64-13.30) | 14.69 (14.34-15.05) | 16.34 (15.79-16.89) | 17.67 (17.09-18.24) | 16.03 (15.46-16.61) |
| Unknown | 2.75 (2.70-2.79) | 3.22 (3.12-3.33) | 2.68 (2.57-2.79) | 2.91 (2.80-3.03) | 2.84 (2.72-2.95) | 2.74 (2.62-2.87) | 2.40 (2.24-2.55) | 2.41 (2.24-2.57) | 2.52 (2.36-2.68) |
| **School performance, weighted (95% CI)** | | | | | | | | | |
| Low (0-19 percentile) | 10.81 (10.72-10.90) | 12.27 (12.09-12.45) | 12.77 (12.54-13.00) | 10.87 (10.66-11.09) | 10.08 (9.86-10.29) | 9.82 (9.60-10.04) | 10.02 (9.68-10.37) | 9.87 (9.53-10.21) | 9.43 (9.09-9.76) |
| Lower-middle (20-39 percentile) | 23.46 (23.33-23.58) | 25.54 (25.28-25.81) | 25.22 (24.93-25.51) | 23.83 (23.54-24.12) | 22.64 (22.34-22.95) | 22.02 (21.71-22.33) | 22.99 (22.49-23.48) | 21.98 (21.55-22.42) | 21.75 (21.26-22.24) |
| Middle (40-59 percentile) | 28.77 (28.65-28.89) | 26.94 (26.69-27.19) | 27.43 (27.16-27.70) | 27.95 (27.67-28.24) | 28.67 (28.37-28.97) | 29.74 (29.43-30.05) | 30.13 (29.68-30.58) | 31.01 (30.55-31.46) | 30.02 (29.55-30.49) |
| Upper-middle (60-79 percentile) | 24.64 (24.52-24.76) | 23.97 (23.74-24.20) | 23.76 (23.48-24.03) | 24.93 (24.65-25.20) | 25.32 (25.04-25.61) | 25.17 (24.87-25.47) | 24.63 (24.17-25.10) | 24.46 (23.99-24.93) | 25.32 (24.86-25.79) |
| High (80-100 percentile) | 12.32 (12.21-12.43) | 11.28 (11.05-11.51) | 10.83 (10.58-11.07) | 12.41 (12.14-12.68) | 13.29 (13.00-13.58) | 13.25 (12.97-13.53) | 12.23 (11.79-12.67) | 12.68 (12.30-13.06) | 13.48 (13.02-13.93) |
| **Parent's highest educational level, weighted (95% CI)** | | | | | | | | | |
| Middle school graduated or under | 20.87 (20.68-21.07) | 10.92 (10.67-11.17) | 12.04 (11.73-12.35) | 13.67 (13.33-14.01) | 14.00 (13.67-14.34) | 28.62 (28.04-29.20) | 33.81 (33.00-34.63) | 34.60 (33.79-35.41) | 30.98 (30.23-31.73) |
| High school graduated | 1.42 (1.39-1.46) | 3.27 (3.16-3.39) | 2.11 (2.00-2.21) | 1.50 (1.42-1.59) | 1.04 (0.98-1.11) | 0.72 (0.67-0.78) | 0.57 (0.50-0.64) | 0.51 (0.44-0.57) | 0.56 (0.49-0.64) |
| University graduated or higher | 24.16 (23.93-24.39) | 36.25 (35.66-36.83) | 33.41 (32.81-34.02) | 28.20 (27.61-28.78) | 25.04 (24.46-25.61) | 17.81 (17.33-18.29) | 15.08 (14.47-15.68) | 13.73 (13.16-14.31) | 12.86 (12.26-13.47) |
| Unknown | 53.54 (53.25-53.84) | 49.56 (48.85-50.27) | 52.44 (51.66-53.22) | 56.63 (55.85-57.41) | 59.92 (59.13-60.70) | 52.84 (52.11-53.58) | 50.55 (49.58-51.51) | 51.16 (50.18-52.13) | 55.59 (54.62-56.56) |
| **Household income, weighted (95% CI)** | | | | | | | | | |
| Middle lower or lower | 16.26 (16.12-16.40) | 23.56 (23.20-23.93) | 21.44 (21.03-21.85) | 17.50 (17.14-17.87) | 14.83 (14.51-15.16) | 12.86 (12.56-13.15) | 12.60 (12.19-13.02) | 10.90 (10.51-11.29) | 10.71 (10.31-11.11) |
| Middle | 47.11 (46.94-47.29) | 46.75 (46.41-47.09) | 47.05 (46.68-47.42) | 47.53 (47.12-47.94) | 46.44 (45.98-46.89) | 46.87 (46.43-47.31) | 47.52 (46.81-48.22) | 48.96 (48.25-49.66) | 46.01 (45.26-46.75) |
| Middle higher or higher | 36.62 (36.38-36.87) | 29.69 (29.20-30.18) | 31.51 (30.97-32.06) | 34.96 (34.39-35.54) | 38.73 (38.13-39.32) | 40.28 (39.72-40.83) | 39.88 (38.99-40.76) | 40.15 (39.25-41.04) | 43.28 (42.35-44.21) |
| **Alcohol consumption, weighted (95% CI)** | | | | | | | | | |
| Non-drinker | 84.35 (84.19-84.51) | 79.08 (78.70-79.46) | 82.15 (81.70-82.60) | 83.28 (82.86-83.70) | 84.50 (84.09-84.91) | 84.03 (83.63-84.43) | 89.34 (88.89-89.78) | 89.27 (88.80-89.73) | 86.95 (86.42-87.48) |
| 1-2 day | 9.13 (9.04-9.23) | 11.75 (11.53-11.96) | 10.23 (9.98-10.48) | 9.73 (9.48-9.97) | 9.27 (9.02-9.52) | 9.09 (8.85-9.34) | 6.34 (6.06-6.63) | 6.52 (6.22-6.82) | 8.19 (7.81-8.57) |
| 3-5 day | 2.95 (2.90-3.00) | 3.80 (3.68-3.93) | 3.49 (3.35-3.63) | 3.28 (3.15-3.42) | 3.05 (2.92-3.19) | 3.17 (3.04-3.31) | 1.93 (1.79-2.07) | 1.88 (1.74-2.03) | 2.18 (2.03-2.33) |
| 6-9 day | 1.66 (1.62-1.69) | 2.39 (2.30-2.49) | 1.92 (1.82-2.02) | 1.74 (1.64-1.84) | 1.49 (1.40-1.59) | 1.71 (1.62-1.81) | 1.13 (1.02-1.24) | 1.07 (0.96-1.19) | 1.32 (1.19-1.45) |
| ≥ 10 day | 1.91 (1.87-1.95) | 2.98 (2.86-3.09) | 2.20 (2.09-2.32) | 1.97 (1.87-2.07) | 1.68 (1.58-1.78) | 1.99 (1.88-2.10) | 1.26 (1.12-1.39) | 1.25 (1.13-1.37) | 1.36 (1.24-1.48) |
| **Smoking status, weighted (95% CI)** | | | | | | | | | |
| Non-smoker | 83.38 (83.18-83.58) | 73.45 (72.95-73.94) | 77.01 (76.41-77.61) | 81.30 (80.71-81.89) | 85.69 (85.17-86.21) | 86.16 (85.69-86.62) | 89.79 (89.26-90.32) | 90.11 (89.62-90.60) | 91.14 (90.67-91.62) |
| Smoker | 16.62 (16.42-16.82) | 26.55 (26.06-27.05) | 22.99 (22.39-23.59) | 18.70 (18.11-19.29) | 14.31 (13.79-14.83) | 13.84 (13.38-14.31) | 10.21 (9.68-10.74) | 9.89 (9.40-10.38) | 8.86 (8.38-9.33) |
| **Stress status, weighted (95% CI)^b^** | | | | | | | | | |
| None | 3.18 (3.13-3.23) | 2.29 (2.19-2.38) | 2.81 (2.70-2.93) | 3.61 (3.48-3.75) | 3.76 (3.61-3.91) | 3.62 (3.47-3.76) | 3.59 (3.40-3.78) | 3.20 (3.02-3.39) | 2.86 (2.68-3.03) |
| Mild | 15.26 (15.15-15.37) | 13.52 (13.32-13.73) | 14.16 (13.91-14.41) | 16.58 (16.31-16.85) | 16.21 (15.92-16.50) | 15.09 (14.79-15.39) | 17.80 (17.35-18.25) | 15.47 (15.08-15.87) | 13.94 (13.58-14.31) |
| Moderate | 42.30 (42.16-42.43) | 41.15 (40.86-41.44) | 41.38 (41.05-41.70) | 43.60 (43.29-43.91) | 42.75 (42.43-43.08) | 41.11 (40.75-41.46) | 44.46 (43.98-44.94) | 42.57 (42.07-43.07) | 41.87 (41.36-42.39) |
| High | 28.35 (28.21-28.48) | 30.46 (30.17-30.76) | 30.05 (29.71-30.39) | 26.91 (26.58-27.24) | 27.12 (26.76-27.48) | 28.49 (28.10-28.88) | 25.86 (25.36-26.37) | 27.81 (27.32-28.30) | 29.01 (28.54-29.47) |
| Severe | 10.92 (10.82-11.01) | 12.58 (12.37-12.78) | 11.60 (11.36-11.84) | 9.29 (9.10-9.49) | 10.15 (9.92-10.39) | 11.69 (11.43-11.96) | 8.29 (7.97-8.61) | 10.95 (10.59-11.30) | 12.32 (11.95-12.69) |
| **Sadness and despair, weighted (95% CI)** | | | | | | | | | |
| No | 71.48 (71.33-71.63) | 64.09 (63.76-64.42) | 69.33 (68.95-69.71) | 74.83 (74.50-75.15) | 74.71 (74.35-75.07) | 72.39 (71.98-72.80) | 74.81 (74.25-75.36) | 73.22 (72.69-73.75) | 71.28 (70.74-71.82) |
| Yes | 28.52 (28.37-28.67) | 35.91 (35.58-36.24) | 30.67 (30.29-31.05) | 25.17 (24.85-25.50) | 25.29 (24.93-25.65) | 27.61 (27.20-28.02) | 25.19 (24.64-25.75) | 26.78 (26.25-27.31) | 28.72 (28.18-29.26) |
| **Suicidal thoughts, weighted (95% CI)** | | | | | | | | | |
| No | 85.63 (85.52-85.74) | 80.65 (80.37-80.93) | 82.57 (82.27-82.86) | 87.62 (87.39-87.85) | 87.89 (87.64-88.15) | 86.78 (86.50-87.06) | 89.12 (88.75-89.49) | 87.29 (86.90-87.68) | 85.73 (85.31-86.15) |
| Yes | 14.37 (14.26-14.48) | 19.35 (19.07-19.63) | 17.43 (17.14-17.73) | 12.38 (12.15-12.61) | 12.11 (11.85-12.36) | 13.22 (12.94-13.50) | 10.88 (10.51-11.25) | 12.71 (12.32-13.10) | 14.27 (13.85-14.69) |
| **Suicidal attempts, weighted (95% CI)** | | | | | | | | | |
| No | 96.92 (96.87-96.96) | 95.38 (95.26-95.51) | 95.91 (95.78-96.05) | 97.32 (97.21-97.42) | 97.53 (97.42-97.63) | 96.98 (96.86-97.11) | 97.98 (97.84-98.11) | 97.79 (97.65-97.94) | 97.35 (97.18-97.52) |
| Yes | 3.08 (3.04-3.13) | 4.62 (4.49-4.74) | 4.09 (3.95-4.22) | 2.68 (2.58-2.79) | 2.47 (2.37-2.58) | 3.02 (2.89-3.14) | 2.02 (1.89-2.16) | 2.21 (2.06-2.35) | 2.65 (2.48-2.82) |

* Abbreviation: BMI, body mass index; KYRBS, Korea Youth Risk Behavior Web-based Survey.

^a^ According to Asia-Pacific guidelines, BMI is divided into 4 groups: underweight (<18.5 kg/m^2^), normal (18.5-22.9 kg/m^2^), overweight (23.0-24.9 kg/m^2^), and obese (25.0 kg/m^2^).

^b^ Stress was defined by receipt of mental health counseling owing to stress.

**Table S2.** Demographic characteristics of AD among excluded and included participants.

| **Characteristics** | Overall | Atopic dermatitis | Non-atopic dermatitis |
| --- | --- | --- | --- |
| **Overall, n** | 917,461 | 62,075 (6.68) | 855,386 (93.32) |
| **Grade, n (%)** |  |  |  |
| 7th–9th grade (middle school) | 468,443 | 31,023 (6.49) | 437,420 (93.51) |
| 10th–12th grade (high school) | 449,018 | 31,052 (6.85) | 417,966 (93.15) |
| **Sex, n (%)** |  |  |  |
| Male | 471,306 | 26,939 (5.68) | 444,367 (94.32) |
| Female | 446,155 | 35,136 (7.76) | 411,019 (92.24) |
| **Region of residence, n (%)** |  |  |  |
| Urban | 415,042 | 27,804 (6.63) | 387,238 (93.37) |
| Rural | 502,419 | 34,271 (6.71) | 468,148 (93.29) |
| **BMI group, n (%)^a^** |  |  |  |
| Underweight | 214,927 | 12,885 (5.92) | 202,042 (94.08) |
| Normal | 465,651 | 31,358 (6.65) | 434,293 (93.35) |
| Overweight | 103,463 | 7,655 (7.22) | 95,808 (92.78) |
| Obese | 106,900 | 7,886 (7.21) | 99,014 (92.79) |
| Unknown | 26,520 | 2,291 (8.59) | 24,229 (91.41) |
| **School performance, n (%)** |  |  |  |
| Low (0-19 percentile) | 102,336 | 7,530 (7.26) | 94,806 (92.74) |
| Lower-middle (20-39 percentile) | 218,304 | 15,095 (6.79) | 203,209 (93.21) |
| Middle (40-59 percentile) | 259,529 | 16,904 (6.44) | 242,625 (93.56) |
| Upper-middle (60-79 percentile) | 225,194 | 15,035 (6.58) | 210,159 (93.42) |
| High (80-100 percentile) | 112,098 | 7,511 (6.68) | 104,587 (93.32) |
| **Parent's highest educational level, n (%)** |  |  |  |
| Middle school graduated or under | 173,256 | 10,110 (5.79) | 163,146 (94.21) |
| High school graduated | 17,879 | 1,190 (6.96) | 16,689 (93.04) |
| University graduated or higher | 253,762 | 17,588 (6.95) | 236,174 (93.05) |
| Unknown | 472,564 | 33,187 (6.89) | 439,377 (93.11) |
| **Household income, n (%)** |  |  |  |
| Middle lower or lower | 165,287 | 12,848 (7.86) | 152,439 (92.14) |
| Middle | 434,364 | 28,556 (6.50) | 405,808 (93.50) |
| Middle higher or higher | 317,810 | 20,671 (6.38) | 297,139 (93.62) |
| **Alcohol** **consumption, n (%)** |  |  |  |
| Non-drinker | 765,939 | 50,474 (6.51) | 715,465 (93.49) |
| 1-2 day | 87,842 | 6,386 (7.12) | 81,456 (92.88) |
| 3-5 day | 28,517 | 2,196 (7.64) | 26,321 (92.36) |
| 6-9 day | 16,184 | 1,220 (7.54) | 14,964 (92.46) |
| ≥ 10 day | 18,979 | 1,799 (9.65) | 17,180 (90.35) |
| **Smoking status, n (%)** |  |  |  |
| Non-smoker | 749,053 | 50,037 (6.58) | 699,016 (93.42) |
| Smoker | 168,408 | 12,038 (7.17) | 156,370 (92.83) |
| **Stress status, n (%)^b^** |  |  |  |
| None | 28,795 | 1,531 (5.29) | 27,264 (94.71) |
| Mild | 139,224 | 7,105 (5.08) | 132,119 (94.92) |
| Moderate | 384,391 | 24,112 (6.19) | 360,279 (93.81) |
| High | 262,510 | 19,894 (7.50) | 242,616 (92.50) |
| Severe | 102,541 | 9,433 (9.06) | 93,108 (90.94) |
| **Sadness and despair, n (%)** |  |  |  |
| No | 648,540 | 39,440 (6.03) | 609,100 (93.97) |
| Yes | 268,921 | 22,635 (8.30) | 246,286 (91.70) |
| **Suicidal thoughts, n (%)** |  |  |  |
| No | 779,924 | 49,814 (6.32) | 730,110 (93.68) |
| Yes | 137,537 | 12,261 (8.77) | 125,276 (91.23) |
| **Suicidal attempts, n (%)** |  |  |  |
| No | 886,696 | 58,881 (6.56) | 827,815 (93.44) |
| Yes | 30,765 | 3,194 (10.32) | 27,571 (89.68) |

* Abbreviation: AD, atopic dermatitis; BMI, body mass index.

^a^ According to Asia-Pacific guidelines, BMI is divided into 4 groups: underweight (<18.5 kg/m^2^), normal (18.5-22.9 kg/m^2^), overweight (23.0-24.9 kg/m^2^), and obese (25.0 kg/m^2^).

^b^ Stress was defined by receipt of mental health counseling owing to stress.

**Table S3**. Ratio of ORs for association between prevalence of AD and each socioeconomic factor, 2020-2022

|  | 2020-2022; weighted OR (95% CI) | 2020; weighted OR (95% CI) | 2021; weighted OR (95% CI) | 2022; weighted OR (95% CI) | Ratio of ORs (2020/2021); weighted ratio of OR (95% CI) | Ratio of ORs (2021/2022); weighted ratio of OR (95% CI) |  |
| --- | --- | --- | --- | --- | --- | --- | --- |
|  |  |  |  |  |  |  |  |
|  |  |  |  |  |  |  |  |
|  |  |  |  |  |  |  |  |
| **Grade, weighted (95% CI)** | | | | | | |  |
| 7th–9th grade (middle school) | 1.00 (reference) | 1.00 (reference) | 1.00 (reference) | 1.00 (reference) | 1.00 (reference) | 1.00 (reference) |  |
| 10th–12th grade (high school) | **1.11 (1.06 to 1.16)** | **1.08 (1.00 to 1.16)** | **1.15 (1.07 to 1.24)** | **1.10 (1.01 to 1.19)** | 1.07 (0.96 to 1.18) | 0.96 (0.86 to 1.07) |  |
| **Sex, weighted (95% CI)** | | | | | | |  |
| Male | 1.00 (reference) | 1.00 (reference) | 1.00 (reference) | 1.00 (reference) | 1.00 (reference) | 1.00 (reference) |  |
| Female | **1.38 (1.32 to 1.44)** | **1.40 (1.31 to 1.51)** | **1.37 (1.27 to 1.48)** | **1.36 (1.26 to 1.47)** | 0.98 (0.88 to 1.09) | 0.99 (0.89 to 1.11) |  |
| **Region of residence, weighted (95% CI)** | | | | | | |  |
| Rural | 1.00 (reference) | 1.00 (reference) | 1.00 (reference) | 1.00 (reference) | 1.00 (reference) | 1.00 (reference) |  |
| Urban | 1.02 (0.98 to 1.07) | 0.99 (0.92 to 1.07) | 0.99 (0.92 to 1.07) | **1.09 (1.00 to 1.18)** | 1.00 (0.90 to 1.11) | 1.10 (0.98 to 1.23) |  |
| **BMI group, weighted (95% CI)^a^** | | | | | | |  |
| Underweight | 1.00 (reference) | 1.00 (reference) | 1.00 (reference) | 1.00 (reference) | 1.00 (reference) | 1.00 (reference) |  |
| Normal | **1.11 (1.05 to 1.18)** | **1.11 (1.00 to 1.23)** | **1.16 (1.06 to 1.28)** | 1.05 (0.94 to 1.16) | 1.05 (0.91 to 1.20) | 0.91 (0.79 to 1.04) |  |
| Overweight | **1.15 (1.06 to 1.25)** | 1.14 (0.99 to 1.30) | 1.14 (0.99 to 1.31) | **1.17 (1.02 to 1.35)** | 1.00 (0.82 to 1.22) | 1.03 (0.84 to 1.25) |  |
| Obese | **1.18 (1.10 to 1.27)** | **1.19 (1.05 to 1.35)** | **1.20 (1.06 to 1.36)** | 1.13 (0.99 to 1.29) | 1.01 (0.85 to 1.20) | 0.94 (0.79 to 1.13) |  |
| **School performance, weighted (95% CI)** | | | | | | |  |
| Middle (40-59 percentile) | 1.00 (reference) | 1.00 (reference) | 1.00 (reference) | 1.00 (reference) | 1.00 (reference) | 1.00 (reference) |  |
| Low (0-19 percentile) | **1.13 (1.05 to 1.23)** | 1.04 (0.91 to 1.20) | 1.11 (0.97 to 1.27) | **1.27 (1.11 to 1.45)** | 1.07 (0.88 to 1.29) | 1.14 (0.95 to 1.38) |  |
| Lower-middle (20-39 percentile) | 1.01 (0.94 to 1.07) | 1.01 (0.91 to 1.12) | 1.03 (0.92 to 1.15) | 0.97 (0.86 to 1.10) | 1.02 (0.88 to 1.19) | 0.94 (0.80 to 1.11) |  |
| Upper-middle (60-79 percentile) | 1.01 (0.95 to 1.07) | 0.97 (0.88 to 1.08) | 1.01 (0.90 to 1.12) | 1.05 (0.94 to 1.17) | 1.04 (0.90 to 1.21) | 1.04 (0.89 to 1.21) |  |
| High (80-100 percentile) | 1.04 (0.96 to 1.12) | 1.03 (0.90 to 1.17) | 1.01 (0.88 to 1.15) | 1.09 (0.96 to 1.24) | 0.98 (0.81 to 1.18) | 1.08 (0.90 to 1.30) |  |
| **Parent's highest educational level, weighted (95% CI)** | | | | | | |  |
| Middle school graduated or under | 1.00 (reference) | 1.00 (reference) | 1.00 (reference) | 1.00 (reference) | 1.00 (reference) | 1.00 (reference) |  |
| High school graduated | **1.41 (1.08 to 1.84)** | 1.20 (0.75 to 1.92) | 1.38 (0.85 to 2.23) | **1.64 (1.07 to 2.53)** | 1.15 (0.59 to 2.26) | 1.19 (0.62 to 2.27) |  |
| University graduated or higher | **1.19 (1.11 to 1.27)** | **1.28 (1.15 to 1.43)** | **1.30 (1.16 to 1.45)** | 0.97 (0.85 to 1.10) | 1.02 (0.87 to 1.19) | **0.75 (0.63 to 0.89)** |  |
| **Household income, weighted (95% CI)** | | | | | | |  |
| Middle lower or lower | 1.00 (reference) | 1.00 (reference) | 1.00 (reference) | 1.00 (reference) | 1.00 (reference) | 1.00 (reference) |  |
| Middle | **1.39 (1.30 to 1.49)** | **1.29 (1.15 to 1.46)** | **1.52 (1.34 to 1.72)** | **1.38 (1.23 to 1.54)** | 1.18 (0.99 to 1.40) | 0.91 (0.77 to 1.07) |  |
| Middle higher or higher | 1.01 (0.96 to 1.06) | 0.95 (0.88 to 1.03) | 1.10 (1.02 to 1.19) | 0.97 (0.89 to 1.07) | **1.16 (1.04 to 1.29)** | **0.88 (0.78 to 1.00)** |  |
| **Alcohol consumption, weighted (95% CI)** | | | | | | |  |
| Non-drinker | 1.00 (reference) | 1.00 (reference) | 1.00 (reference) | 1.00 (reference) | 1.00 (reference) | 1.00 (reference) |  |
| 1-2 day | **1.10 (1.01 to 1.20)** | **1.19 (1.04 to 1.37)** | 1.03 (0.88 to 1.20) | 1.11 (0.95 to 1.29) | 0.87 (0.70 to 1.07) | 1.08 (0.87 to 1.34) |  |
| 3-5 day | **1.26 (1.09 to 1.46)** | 1.09 (0.84 to 1.41) | **1.42 (1.10 to 1.83)** | **1.30 (1.00 to 1.67)** | 1.30 (0.91 to 1.87) | 0.92 (0.64 to 1.31) |  |
| 6-9 day | **1.26 (1.03 to 1.54)** | 1.23 (0.89 to 1.68) | 1.36 (0.93 to 2.01) | 1.22 (0.87 to 1.70) | 1.11 (0.67 to 1.82) | 0.90 (0.54 to 1.50) |  |
| ≥ 10 day | **1.80 (1.52 to 2.12)** | **1.60 (1.21 to 2.13)** | **1.95 (1.48 to 2.57)** | **1.86 (1.38 to 2.51)** | 1.22 (0.82 to 1.81) | 0.95 (0.64 to 1.43) |  |
| **Smoking status, weighted (95% CI)** | | | | | | |  |
| Non-smoker | 1.00 (reference) | 1.00 (reference) | 1.00 (reference) | 1.00 (reference) | 1.00 (reference) | 1.00 (reference) |  |
| Smoker | **1.17 (1.09 to 1.26)** | 1.09 (0.97 to 1.23) | **1.20 (1.06 to 1.36)** | **1.24 (1.09 to 1.43)** | 1.10 (0.93 to 1.31) | 1.03 (0.86 to 1.24) |  |
| **Stress status, weighted (95% CI)^b^** | | | | | | |  |
| None | 1.00 (reference) | 1.00 (reference) | 1.00 (reference) | 1.00 (reference) | 1.00 (reference) | 1.00 (reference) |  |
| Mild | 1.01 (0.87 to 1.17) | 1.15 (0.91 to 1.46) | 0.87 (0.67 to 1.12) | 1.00 (0.75 to 1.33) | 0.76 (0.53 to 1.07) | 1.15 (0.78 to 1.69) |  |
| Moderate | **1.22 (1.06 to 1.40)** | **1.40 (1.12 to 1.76)** | **1.13 (0.89 to 1.45)** | 1.12 (0.85 to 1.46) | 0.81 (0.58 to 1.13) | 0.99 (0.69 to 1.43) |  |
| High | **1.51 (1.31 to 1.74)** | **1.68 (1.34 to 2.11)** | **1.45 (1.14 to 1.85)** | **1.42 (1.08 to 1.86)** | 0.86 (0.62 to 1.20) | 0.98 (0.68 to 1.41) |  |
| Severe | **1.84 (1.59 to 2.14)** | **2.03 (1.58 to 2.61)** | **1.66 (1.30 to 2.13)** | **1.88 (1.42 to 2.48)** | 0.82 (0.58 to 1.16) | 1.13 (0.78 to 1.64) |  |
| **Sadness and despair, weighted (95% CI)** | | | | | | |  |
| No | 1.00 (reference) | 1.00 (reference) | 1.00 (reference) | 1.00 (reference) | 1.00 (reference) | 1.00 (reference) |  |
| Yes | **1.39 (1.33 to 1.46)** | **1.36 (1.26 to 1.48)** | **1.50 (1.39 to 1.62)** | **1.33 (1.22 to 1.44)** | 1.10 (0.99 to 1.23) | **0.89 (0.79 to 0.99)** |  |
| **Suicidal thoughts, weighted (95% CI)** | | | | | | |  |
| No | 1.00 (reference) | 1.00 (reference) | 1.00 (reference) | 1.00 (reference) | 1.00 (reference) | 1.00 (reference) |  |
| Yes | **1.46 (1.37 to 1.55)** | **1.34 (1.19 to 1.51)** | **1.59 (1.45 to 1.75)** | **1.46 (1.31 to 1.62)** | **1.19 (1.02 to 1.38)** | 0.92 (0.80 to 1.06) |  |
| **Suicidal attempts, weighted (95% CI)** | | | | | | |  |
| No | 1.00 (reference) | 1.00 (reference) | 1.00 (reference) | 1.00 (reference) | 1.00 (reference) | 1.00 (reference) |  |
| Yes | **1.77 (1.57 to 2.00)** | **1.38 (1.08 to 1.78)** | **1.76 (1.45 to 2.13)** | **2.17 (1.80 to 2.63)** | 1.28 (0.93 to 1.75) | 1.23 (0.94 to 1.62) |  |

* Abbreviation: AD, atopic dermatitis; BMI, body mass index; CI, confidence interval; OR, odds ratio.

^a^ According to Asia-Pacific guidelines, BMI is divided into 4 groups: underweight (<18.5 kg/m^2^), normal (18.5-22.9 kg/m^2^), overweight (23.0-24.9 kg/m^2^), and obese (25.0 kg/m^2^).

^b^ Stress was defined by receipt of mental health counseling owing to stress.
